# Intrinsic Activity in the Fly Brain Gates Visual Information during Behavioral Choices

**DOI:** 10.1371/journal.pone.0014455

**Published:** 2010-12-30

**Authors:** Shiming Tang, Mikko Juusola

**Affiliations:** 1 State Key Laboratory of Cognitive Neuroscience and Learning, Beijing Normal University, Beijing, China; 2 State Key Laboratory of Brain and Cognitive Science, Institute of Biophysics, Chinese Academy of Sciences, Beijing, China; 3 Department of Biomedical Science, University of Sheffield, Sheffield, United Kingdom; Freie Universität Berlin, Germany

## Abstract

The small insect brain is often described as an input/output system that executes reflex-like behaviors. It can also initiate neural activity and behaviors intrinsically, seen as spontaneous behaviors, different arousal states and sleep. However, less is known about how intrinsic activity in neural circuits affects sensory information processing in the insect brain and variability in behavior. Here, by simultaneously monitoring *Drosophila*'s behavioral choices and brain activity in a flight simulator system, we identify intrinsic activity that is associated with the act of selecting between visual stimuli. We recorded neural output (multiunit action potentials and local field potentials) in the left and right optic lobes of a tethered flying *Drosophila*, while its attempts to follow visual motion (yaw torque) were measured by a torque meter. We show that when facing competing motion stimuli on its left and right, *Drosophila* typically generate large torque responses that flip from side to side. The delayed onset (0.1–1 s) and spontaneous switch-like dynamics of these responses, and the fact that the flies sometimes oppose the stimuli by flying straight, make this behavior different from the classic steering reflexes. *Drosophila*, thus, seem to *choose* one stimulus at a time and attempt to rotate toward its direction. With this behavior, the neural output of the optic lobes alternates; being augmented on the side chosen for body rotation and suppressed on the opposite side, even though the visual input to the fly eyes stays the same. Thus, the flow of information from the fly eyes is gated intrinsically. Such modulation can be noise-induced or intentional; with one possibility being that the fly brain highlights chosen information while ignoring the irrelevant, similar to what we know to occur in higher animals.

## Introduction

By evolving elaborate patterns of behavior, insects have conquered myriads of terrains. Adaptations in the behaviors to ongoing environmental changes further contribute to their success. Perhaps not surprisingly, an insect can react to the same cue quite differently. Although the mechanisms of this behavioral variability are not understood, it is likely to denote variability in the neural information processing, from sensors to effectors, and any factors between them [1]–[4]. Such factors can be noise [5], recall of previous encounters with similar cues (adaptation, learning or memory) [6]–[8], fatigue or change in behavioral or arousal states [9]–[17], or it can arise spontaneously from circuits' rhythmic or nonlinear dynamics [18]–[23], named as intrinsic activity in contrast to activity evoked by external stimuli. The problem is that by observing an insect's reactions alone, it is very difficult, if not impossible, to deduce the neural basis for the change in its behavior.

Here, we set out to examine how intrinsic activity within the small brain of *Drosophila* affects the flow of information from its eyes, when a fly makes a decision to follow visual motion. In a modified flight simulator system, a tethered flying fly sees two competing motion stimuli (monocular flow fields) of equal strength, one on its left and the other on its right. If it chooses to follow motion (for whatever reason) it can do so only one stimulus at a time. This response (yaw torque toward left or right) is taken as a fly's report for the chosen stimulus, whereas two microelectrodes, implanted in its left and right optic lobes, are used to look for neural signatures (in multiunit action potentials and local field potentials) for this choice. In a sequence of experiments using tethered flies that either rested (to provide baseline signals) or flew, we show that when a *Drosophila* generates a torque response to left or right, the neural activity in the optic lobes is enhanced on the chosen side and suppressed on the opposite side, although visual input to its eyes remains unchanged during this behavior. Our findings, therefore, show that intrinsic neural mechanisms gate visual information processing within the optic lobes, providing new mechanistic insight into the origin of variability in insect behavior. Furthermore, if future studies can establish that this modulation is not induced by noise but intentional, then these results could reveal possible neural correlates for attending (increase in activity) and ignoring (reduction in activity) in the *Drosophila* brain.

## Results

### Measuring Visual Behavior of *Drosophila* during Competing Motion Stimuli

**Figure 1:**
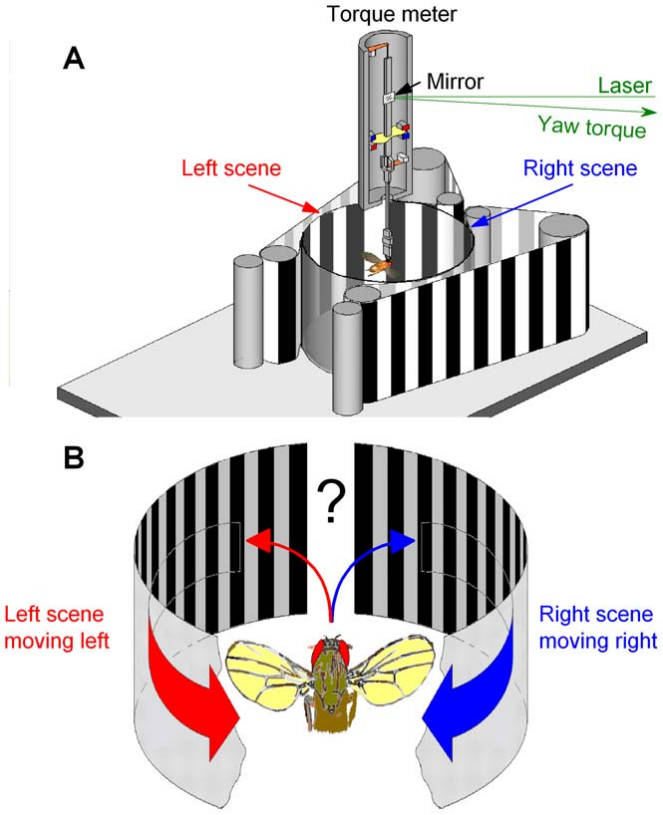
Open loop experiments for measuring a *Drosophila*'s orienting behavior (torque responses) to competing stimuli. (A) Schematic drawing of the flight simulator system. Two identical paper strips, having the same black and white stripe pattern, curve along the surface of a transparent cylinder on the left (red) and right (blue) of a tethered flying fly, thus forming the left and right scenes, respectively. The scenes are moved by an electrical motor. The yaw torque of the fly, *i.e.* its responses toward the moving scenes, is measured by an opto-mechanical torque meter. A small mirror linearly reflects changes in the yaw torque; the light-return of a laser beam over distance greatly amplifies this signal for an optical sensor. (B) Because the fly's head is clamped in a fixed position and orientation, preventing its movements, the fly should see two identical scenes, on its left and right, which simultaneously move to the opposite directions without any overlapping visual fields. Thus, this stimulation generates two isolated monocular flow fields, one for each eye. The fly's torque response indicates which of the two stimuli (moving scenes) it has chosen to pursue at any one time.

We adapted a flight simulator system [7], [16], [24], [25] for *Drosophila* to present competing visual motions (**Figs. 1A**). When a tethered flying fly sees a movement, it will orient (turn) toward it [24], [26]. Although prevented from turning by a torque meter, the fly's efforts produce minute yaw torque signals, whose size and polarity give the strength and direction, respectively, of these attempts [24], [26]. When facing two moving objects, one on its left and the other on its right, *Drosophila* can restrict its torque response to one of them [16], [27]. In attempt to evoke comparable behavior with a stronger stimulus, we expanded the size of the two moving objects to cover large sectors of the left and right hemifields, respectively (**Fig. 1B**).

Importantly, in our flight arena, both the left and the right eye face 150°-wide moving scenes, *i.e.* two monocular flow fields; the frontal and caudal parts of the respective hemifields are blanked to eliminate binocular motion cues that can trigger landing or avoidance responses [28]. This motion stimulation, of using two isolated lateral flow fields, differs from the forward flight [29] or frontal field expansion [25], during which a fly would see a continuous flow field from left to right (see Text S1).

### *Drosophila* Generates Switch-like Torque Responses between Competing Motion Stimuli

In the competing stimuli paradigm, visual information in the left and right vie for the fly's torque responses (competitive selection). A tethered flying fly faces two symmetric scenes of visual patterns (*e.g.* black and white vertical stripes), running in opposite directions, to its left and right. Apart from the two opposing motion vectors, everything else in the two scenes remains equal. Most importantly, visual input to the fly's eyes remains equal even during large responses, because its head is immobilized by the torque meter [24], [26]. The fly cannot make two opposite responses at the same time. In this competitive case, it may choose to react to one direction, generating yaw torque toward (or against) this side, or by balancing its optomotor output, continue in a straight course [16], [24], [29].

**Figure 2:**
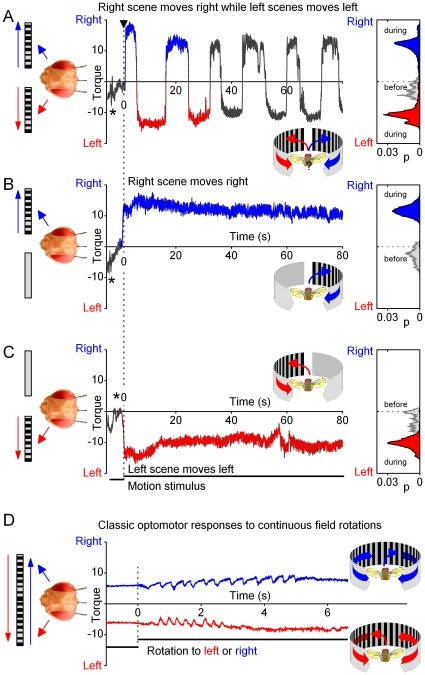
*Drosophila*'s behavior to competing left and right visual motion stimuli. (A) A flying tethered *Drosophila* faces two identical scenes of black and white stripes, one on its left and the other on its right, in a flight simulator system. When the scenes are still, a fly often generates brief saccades (stars), characteristic of normal exploratory behavior [26], but orients mostly straight. When the scenes are set to sweep together to the opposing directions (dotted line, at time zero), a fly's attempts to rotate (yaw torque) toward the left (red trace) or right (blue trace) stimulus begin to flip from side to side with switch-like dynamics, as measured by the torque meter. Throughout these strong responses, the visual input to the fly's eyes remains virtually unchanged, because the fly's head is firmly held by the torque meter in a fixed position. The behavior consists of stereotypical one-sided torque responses, which last 5–15 s, yet their duration and patterning varies greatly from fly to fly (*cf*. Figs. 6, S3 and S6). The torque responses of a *Drosophila* to right (up) or left (down) during bilaterally moving scenes (A) are of similar strength to its responses when the right (B) or left (C) scenes are moved separately. The insets show the corresponding probability density functions before and during the motion stimulation. Thus, with competing stimuli (A), *Drosophila* appears to *choose* one scene at a time and exert its yaw torque according to it, before switching to the opposite stimulus. (D) The classical optomotor responses of a fly look different. Tethered to the same torque meter, a flying fly was exposed to 360° visual field (having similar black and white stripes, as above) that rotated left or right. A fly tries to stabilize its vision by attempting to turn into the same direction as the rotating stimulus. The resulting optomotor responses, which contain correction saccades, are typically evoked from the stimulus onset onwards, characteristic of steering reflexes. They are also much smaller than the torque responses to stimuli in A–C. Note the 10-times briefer time scale in D. The optomotor responses in D are shifted up and down to highlight their waveforms. Torque is in arbitrary units.

When the scenes were still, *Drosophila* generated small recurrent body saccades between left and right (**Fig. 2A**, stars; see also **Figs. S2**, **S3**, **S4**, **S5**, **S6**), similar to exploratory behavior [26]. However, once the scenes started moving (top, black arrow), the flies typically began to generate 2–10-times stronger yaw torque, *i.e*. intense attempts to rotate to right or left. These large torque responses flipped from side to side as if the right and the left movement were presented alternatingly to a fly, when instead both of the scenes were moving together. Because of the two-state nature of this behavior, *Drosophila* seemed to restrict their responses to one side at a time for 3–20 s, until reacting again to the other side. This periodicity varied considerably between individual flies (*cf*. **Fig. S3**), and sometimes also contained epochs of flying straight (**Figs. S3**, **S5** and **S6**). Superimposed on the switch-like motif, the behavior often included smaller saccades (100–300 ms; *cf*. **Figs. S3** and **S6**), possibly as attempts to stabilize, or enhance [30], visual information in the optic flow field from the same direction.

The switch-like torque responses between two motion scenes is a conspicuous behavior, and of course very different from the fast automatic steering reflexes that flying insects use to control their locomotion in changing environments [29], [31], [32]. We, therefore, needed to test its generality in open loop settings by changing optic flow variables in the competing stimuli paradigm. We found that *Drosophila*'s torque responses flipped from side to side with different stimulus speeds (**Fig. S6**), and with patterns of different shapes and sizes (crosses or circles, **Fig. S2D**). Most flies displayed this behavior, sometimes for several minutes. Further experiments, in which a fly was slightly repositioned within the flight arena, or in which we dephased the two stimuli, gave similar results, suggesting that the switch-like responses were unlikely to be evoked by visual asymmetry, or by certain pattern features. See Text S1 for further details.

Apart from noise, there are two basic schemes how the fly brain could initiate the switch-like behavior during competing stimulation. It could either reduce - or increase - the flow of visual input from one eye, or reduce – or increase - the motor output of the flight control system to the opposing stimulus, thereby creating a neural imbalance to drive a torque response to one direction at a time.

### Switch-Like Responses Are not Solely Input-Driven

To gain more insight on these hypotheses, we tested how a tethered flying *Drosophila* responds to a one-sided stimulus. In this monocular stimulus paradigm, the left or right moving scene was sweeping front-to-back, while the other side displayed a motionless blank screen. Interestingly, we found that the initial torque responses toward a single moving scene were of similar size and shape to the responses to the same moving scene in the competing stimuli paradigm (**Figs. 2B–C**). The level of reciprocal symmetry in these responses was analogous to that evoked by uni- or bilaterally oscillating bars [16], [27], suggesting that both of these responses may share a common mechanism of initiation. Furthermore, classical steering reflexes (or optomotor responses) seemed very different (**Fig. 2D**). There, exposed to rotating visual stimuli, a fly tried to stabilize the visual scenery by turning (its head and body; eyes) into the same direction as the rotational stimulus [24] (left or right), evoking spiky responses that were much smaller and briefer than the torque responses in the competing stimuli paradigm.

These results were important for two reasons. First, they implied that the conspicuous switch-like behavior might not be purely input-driven. Otherwise, the responses to a single stimulus would have been stronger without the competing stimulus than with it. Second, because these responses had stereotypical early waveforms in both uni- and bilater stimulus paradigms (*cf*. **Fig. 2A** to **Figs. 2B–C**), neural activity that regulated them during competing stimulation must have originated before any motor commands were sent to the flight control system. This deduction, thus, further suggested that to initiate or facilitate switch-like behavior, the flow of visual input, from the eyes to the fly brain, might be modulated by endogenous processing; in other words, intrinsically.

There were other observations supporting these views. During the competing stimulus paradigm, the switch-like responses were sometimes interrupted with periods of flying straight (**Figs. S3**, **S5** and **S6**; these zero torque sections are indicated by small arrows). This aspect of the behavior cannot result from simple reflex-like optomotor steering but requires further processing. We also observed that in response to one-sided motion stimulus, the flies infrequently exerted yaw torque to the opposite direction, toward the blank motionless screen (**Figs. S7A–B**). This unanticipated behavior showed that even during torque responses, evoked by a powerful monocular motion scene, the flies' reactions were not fully input-driven; a fly could attempt to readjust its body orientation at any stage of the stimulation.

### Onset of Switch-Like Responses Is Delayed and Variable

Thus, our findings increasingly suggested that *Drosophila*'s torque responses to competing motion stimuli are initiated and modulated endogenously. To further examine this hypothesis, we next analyzed the initiation of the switch-like behavior from the stimulus onset. We were particularly interested in the variability of the first responses, because their early time course might give indications how the underlying neural dynamics leading to them differed from those of fast automatic steering reflexes.

**Figure 3:**
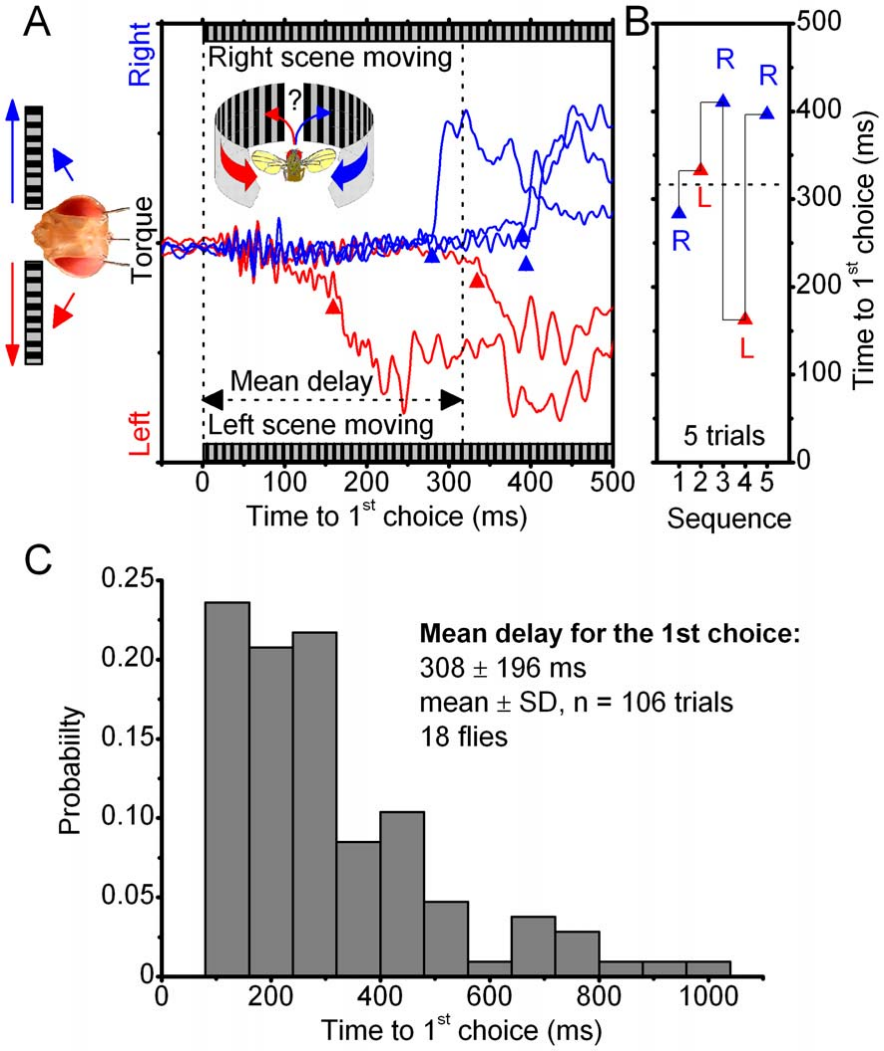
Time-to-choice varies greatly during competing stimulation. (A) A tethered fly is flying in the flight arena, when suddenly the identical scenes on its left and right, are made to move together at the moment of t = 0 (60°/s). It takes on average 316.6±100.4 ms (mean ± SD, n = 5) before the fly begins to react either to the left (red triangle) or right (blue triangle) scene, as measured by time-to-choice of its first switch-like torque responses. The scenes were stopped and started again with tens of seconds between the trials. These orientation responses are highly variable. The double-headed arrow (black) stretches out the mean delay for this fly. (B) Its first responses were either to left (red triangle) or right (blue triangle), showing no side-preference and with time-to-onset, or wait-period, varying from one trial to another (14/18 flies behaved this way). Other flies preferred one stimulus over its counterpart, yet the wait-period for their first switch-like torque response changed greatly between the trials (4/18 flies behaved this way). The experimental settings were kept identical, but the flies “motivation” to perform varied greatly. In the worst case, we could only test this paradigm twice, before the fly lost “interest” and stopped flying. In the best case, the experiment was repeated 20 times. (*C*) Time-to-choice statistics of the flies are skewed with a heavy tail. As there was no real difference in the variable onset between the left and right responses, these results are pooled. Notice, that sometimes it took a fly for over a second to initiate orientation toward its chosen stimulus.

For all the flies tested in the competing stimuli paradigm, we found the initiation of the first response highly variable from trial to trial (**Fig. 3A**). Once the scenes were set in motion (here 60°/s), a fly could wait sometimes for up to seconds (308±196 ms, mean ± SD, n = 106 trials; 18 flies) before exerting decisive yaw torque to its left or right. When the experiment was repeated (after 10–30 s), the same stimulus very often elicited a different response (**Figs. 3B**). The long-tailed distribution of the wait times (**Fig. 3C**), the varying side and dynamics of the first responses implied that these were not rigid steering reflexes toward visual motion or simple avoidance away from it [25] but more complex actions [18]. The reported minimal latency of the so-called *object response* to a single black bar, when moved front-to-back either at 110 or 300°/s, is 35 ms [27], whereas the latency of the collision-avoidance response to object expansion is typically around 50 ms [28]; also, the apparent delays in the steering reflexes to 360° field rotation in **Fig. 2D** seemed similar (53±13 ms; mean ± SD; n = 6 trials). However, in our visual choice paradigm, it took at least another 30–45 ms for a fly to choose the direction of its response, as their shortest wait was 80 ms.

Taken together, the results from the behavioral experiments implied that during continuously moving competing scenes, a *Drosophila* chose one scene at a time and attempted to orient/turn toward it (or away from the other), *i.e. visual selection*. This view is again consistent with an earlier report of *Drosophila*'s switch-like torque responses between bilaterally oscillating bars [16], albeit such stimuli moved differently and covered smaller sections of the eyes than the flow fields used here. However, it remained unclear how *Drosophila* decided upon which scene to choose. Without any neurophysiological evidence of the neural dynamics behind the switches, the behavioral evidence, as it stood here, was only suggestive about the role of the intrinsic activity in decision making. For example, it was still possible that this behavior reflected neural noise. To help to distinguish between different alternatives, we next compared the fly's behavior to the concurrent activity in the left and right optic lobes, picked up by the miniaturized electrodes.

### Measuring Neural Activity in the Optic Lobes

In our experimental set-up, neural activity in the left and right optic lobes of *Drosophila* can be monitored simultaneously with its behavior (torque responses) using miniaturized electrodes (see Materials and Methods). These electrodes can pick-up both firing patterns of nearby neurons, and local field potentials (LFP) that, in case of the very small *Drosophila* brain, seem to signify more global information processing within each optic lobe. For examining how neural activity of the optic lobes might correlate with the fly's behavioral choices, we used the monocular stimulus paradigm for *resting* flies (non flying) and the competing stimuli paradigm for *flying* flies.

### *Resting* Flies: More Activity in the Optic Lobe Facing Movement

**Figure 4:**
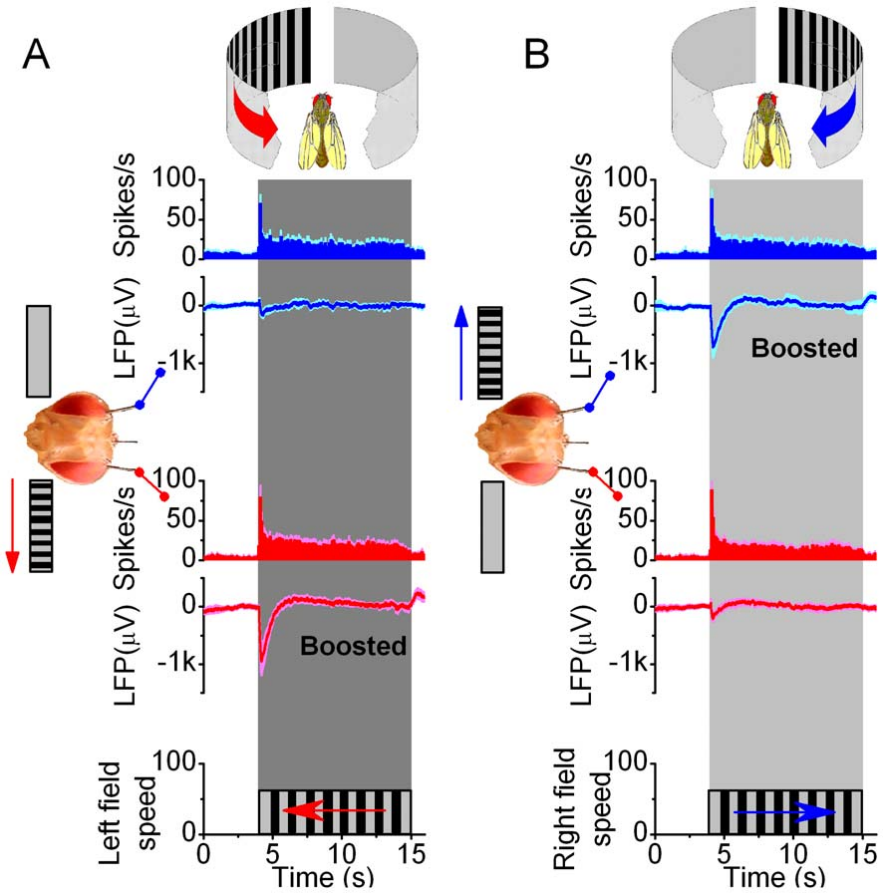
Brain activity increases on the side facing the motion stimulus. Local field potentials (LFPs) in the left and right optic lobes of *resting Drosophila* are enhanced on the side of the moving scene (black and white stripes), whereas the firing patterns show that unilateral visual motion is processed bilaterally in the brain. (A) Neurons in both the right (blue traces) and left (red traces) optic lobes respond simultaneously and adapt rapidly to left motion; this transiently increases their firing rates, amplifying the LFPs. Peak rates: 69.6±29.0 and 79.0±38.0 spikes/s (mean ± SD; right and left electrode, respectively) show no statistical difference, whilst left LFPs are always larger (p = 0.006; ANOVA, one-way Bonferreoni test). (B) Similarly, neurons in both optic lobes respond to right motion. Peak rates: 75.3±28.7 and 87.9±40.4 spikes/s (mean ± SD; right and left electrode, respectively) do not differ statistically, but the right LFPs are always larger (p = 0.012, ANOVA, one-way Bonferreoni test). Without motion stimulus the activity is low: 5.2±1.3 spikes/s (mean ± SD; n = 12). The strong motion-sensitivity suggests that the electrodes reside in the lobula plates. Scenes were separately moved for 6–20 times on either side with 5–10 s interstimulus periods; means ± SEMs shown, n = 6 flies.

Experiments with unilateral visual motion in *resting Drosophila* (**Figs. 4A–B**) showed that each optic lobe received and processed information from both eyes, but that the overall neural activity was always higher (boosted LFPs) within the lobe that faced the movement. During the experiments, the flies remained mostly still, as assessed by their zero torque signals, or visual monitoring. Because the chosen recordings contained relatively little spurious activity (see Text S1), they represented a reasonable account of how the outputs of the left and right optic lobes encoded monocular flow fields.

The finding that these neurons fired selectively to visual motion, suggested that the electrodes were either lodged within the neuropiles called the lobula plates or in their vicinity. The lobula plates contain an intricate web of large motion-sensitive neurons [33], lobula plate tangential cells (LPTCs), many of which have binocular receptive fields and rapid adaptation dynamics [34], [35]. The lobula plates are only a few synapses away from the photoreceptors and the flight muscles [36], receive inputs from motion-sensitive elements in both the ipsi- and contralateral eyes [34] and from higher brain centers [37], [38], and participate in gaze control [34], [39]. Based on their importance in visual behavior in dynamic environments, we reasoned that these neuropiles might play a role in intrinsic modulation of incoming visual information. However, given that our electrodes may also reside or pick up activity outside the lobula plates, we call the recording sites more generally the optic lobes.

### *Flying* Flies: Activity of the Optic Lobes Precedes Behavioral Choices

**Figure 5:**
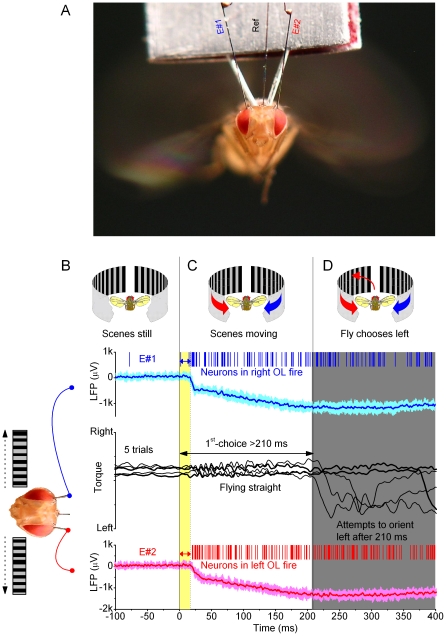
Neural output of the optic lobes to moving stimuli precedes behavioral choices. This figure shows five trials of a single fly in the competing stimuli paradigm. (A) A flying tethered *Drosophila* has three electrodes inserted into its brain: right (E#1) and left (E#2) optic lobes (OL) and reference (Ref). It flies in a flight simulator seeing identical scenes of black and white stripes on its left and right. (B) When the scenes are still, the fly continues flying strength, and the right and left optic lobes show little activity; only a sporadic spike and the local field potentials (LFPs) are flat (E#2, blue traces; E#1 red traces). (C) When the scenes start to sweep to the opposing directions (ft = 0), it takes about 20 ms (yellow) for the optic lobes to respond to these visual stimuli (first spikes, and dips in LFPs). However, the fly still only makes little adjustments in its flight path, *i.e.* the yaw torque remains flat. (D) After minimum of 210 ms of stimulation, the fly finally chooses the left stimulus by attempting to turn left (gray area), seen as intensifying yaw torque (downward). The fly's choice of stimulus (left) is taken from the point where a new clear trajectory starts in the torque response, crossing the midline. The time to 1^st^-choice varies greatly; thick black traces show trials where the fly took 375 and 700 ms to choose the stimulus. In the presented fast time scale, the changes in the yaw torque show no obvious influence on the neural outputs of the optic lobes. Recordings like this imply that the early neural activity in the optic lobes is predominantly evoked by visual motion. Thus, here it appears neither induced by, nor corresponds to, stimulus artifacts or flight muscle activity. LFPs show means ± SDs.

How does the neural activity of the optic lobes represent visual inputs in the competing stimuli paradigm? To begin distinguishing the relevant patters of neural activity involved, we first measured the time from the stimulus onset to the neural response and behavioral choice. Again, a tethered *flying Drosophila* (**Fig. 5A**) was stimulated by identical scenes (*e.g.* stripe patterns) on its left and right, moving to opposing directions. Each switch-like attempt of a fly to turn left or right was then taken as its momentary choice of the stimulus.

Neural activity picked up by the miniaturized electrodes from the optic lobes was typically low when the scenes remained still (**Fig. 5B**). Although the fly's flight muscles were in full action, the electrodes in the optic lobes recorded only sporadic spikes, few and far between, implying stable recording conditions. However, once the scenes were set in motion, it took approximately 15–20 ms (**Fig. 5C**, yellow section) until the electrodes picked up an obvious increase in activity (burst of spikes and hyperpolarizing LFPs), evoked by the visual motion. The delay in these neural responses is consistent with our intracellular measurements of the conduction delays in photoreceptors and primary visual interneurons [40]–[42], and a time estimate of further processing stages leading to the visual motion information arriving to the circuitry in the lobula plate. The baseline activity of the optic lobes remained elevated throughout the competing motion stimulation, and showed little change when finally, after a further 190 ms, the fly chose the left stimulus (by beginning to restrict its torque response to left, **Fig. 5D**).

It is clear from these and other similar recordings that there was neither strong time-dependency nor correlations between the first neural responses of the optic lobes to motion stimuli and the fly's choice of the stimulus. The first neural responses appeared between 12 to 39 ms (1^st^ spike: 20.63±5.14 ms; mean ± SD; 42 optic lobes, 238 trials) from the stimulus onset, while the flies always reported their first choice of stimulus much later, typically after hundreds of ms had passed (*cf*. **Fig. 3C**). Because the exact recording locations of the electrodes within the optic lobes inevitably varied slightly from one lobe to another, so did their sensitivity to pick up neural activity. An observation that one electrode picked up more spikes to the competing stimuli than the other, had obviously nothing to do with a fly's choice of stimulus; thus, neural output of each optic lobe was compared to the torque output separately. However, in the fine time resolution of tens of ms, we failed to find general or consistent interdependencies between the neural outputs and the microstructure of the flies' first switch-like torque responses (*cf*. **Fig. 5D**).

The lack of interdependence between the early motion-elicited neural activity and the time of the first torque response means that (**1**) the processes, which initiate the motor output for choice, require a long integration period, and that (**2**) while gathering more information, these processes seem to exert little impact on the neural outputs of the optic lobes, which thus appear predominantly vision driven.

### *Flying* Flies: Activity of the Optic Lobes Changes with Behavior

**Figure 6:**
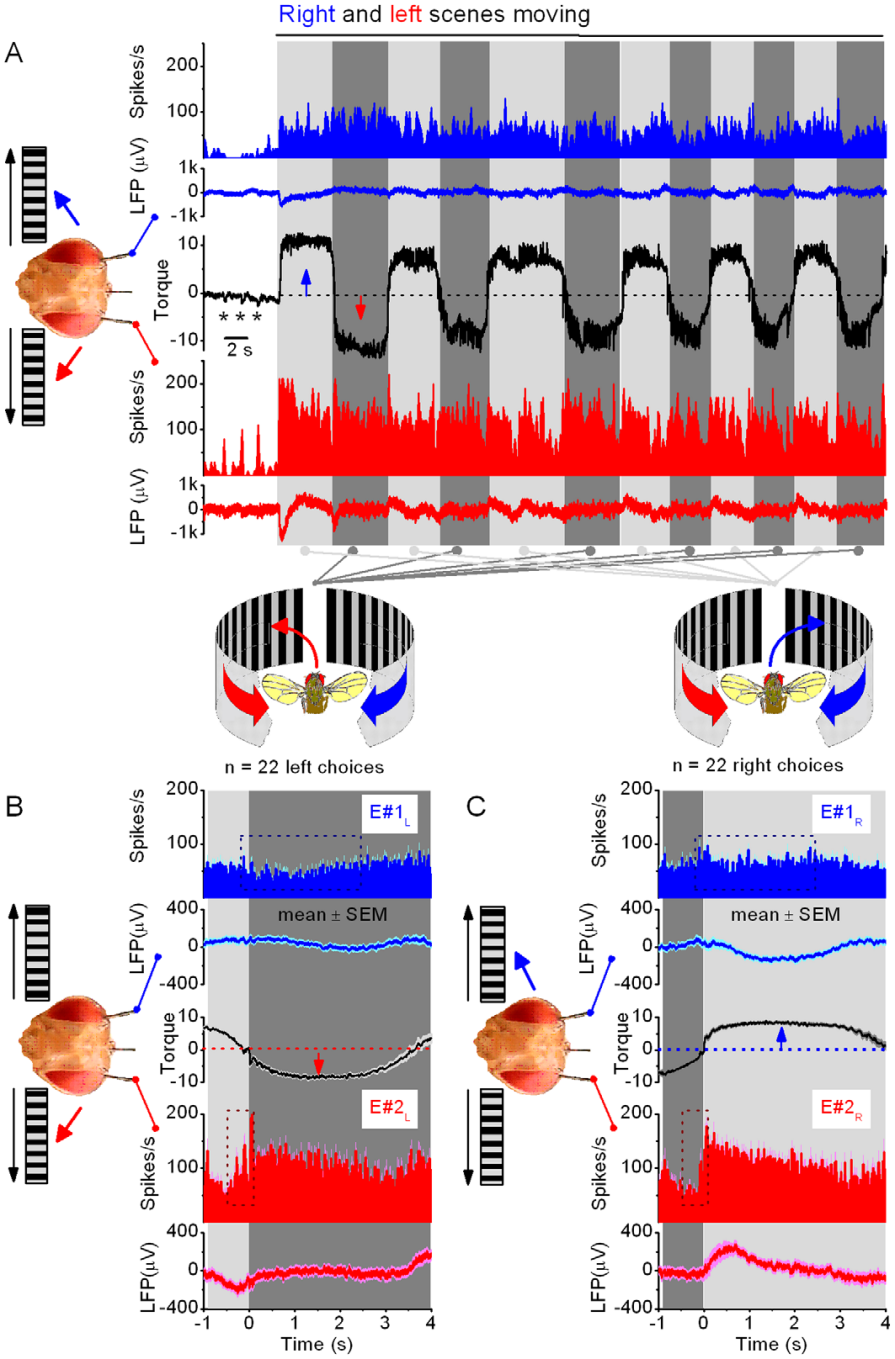
Neural output of the optic lobes is modulated with behavioral choices. (A) A flying tethered *Drosophila* faces identical scenes of black and white stripes on its left and right. When the scenes are still, the fly generates exploratory saccades (stars). When the scenes move to opposing directions, the fly's yaw torque (black) begins eventually to flip between right and left. These behavioral choices of the fly are accompanied with an increased oscillating neural activity both sides of its brain (firing rates and LFPs; blue traces: right electrode, E#1 and red traces: left electrode, E#2). Each choice (or switch-like torque response) can be separated from its neighbors by its clean zero-crossings. Torque responses to right are shown in light gray, and those to left in dark gray. (B–C) show statistics of the neural activity in the left and right LPs for left and right torque responses, respectively (mean ± SEM, n = 22 choices to both directions). The traces were aligned in respect to the corresponding zero-crossings (dotted lines) in the torque signals (black traces). This data was then used for estimating intrinsic modulation (Figs. 7A–B) as the change in the activity of the right optic lobe: E#1 (right torque) – E#1 (left torque); and for the left optic lobe: E#2 (left torque) – E#2 (right torque). For firing rates, the bin-size is 100 ms; torque is in arbitrary units. The dotted boxes in B and C focus on the largest differences in the firing rate in each optic lope for left and right choices.

As it could take hundreds of ms for the fly brain to gather enough information to choose between the two stimuli (*cf.* **Figs. 3C** and **5D**), we expected that possible correlations between neural activity and a fly's orientation choices might emerge gradually or periodically over behaviorally relevant integration times. We therefore looked for such signatures of intrinsic activity, which could signal changes in the accumulation and interpretation of visual information within the fly brain, over prolonged time scales (**Fig. 6**). Owing to the slight sensitivity differences between the electrodes to pick up neural activity, the analysis was naturally done for each optic lobe (*i.e.* electrode) separately.

**Figure 7:**
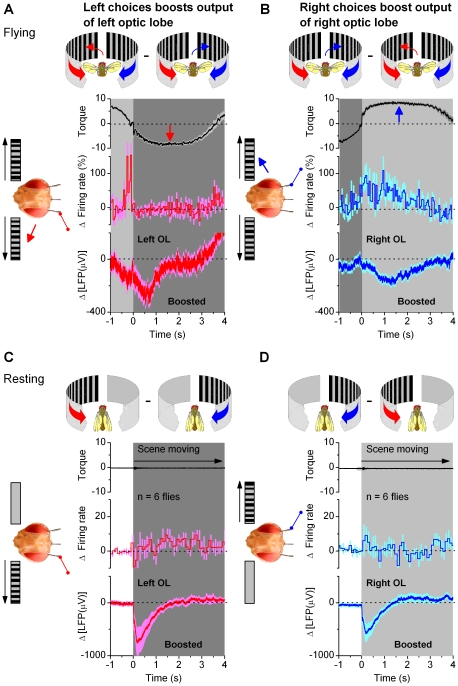
Neural output of the optic lobes increases on the side chosen for torque response. Changes (Δ) in the average transmission of visual motion information are shown for opposing choices (A, B; relative change for firing rates) in flying flies, and for opposing visual stimuli (C, D) in resting flies. Despite seeing equal but opposite motion stimuli (moving scenes of black and white stripes) on its left and right, the activity of the optic lobes changes when a fly chooses the stimulus for its torque response to (A, B) as if the left and right scenes were presented alternatingly to the fly at rest (C, D). (A) Choosing the left stimulus (torque down) boosts the output of the left optic lobe; (B) choosing the right stimulus (up) boosts the output of the right. This data, aligned by the zero-crossings (dotted) in the torque (top) with left/right division (dark/light grey), is from an experiment containing 22 nearly symmetrical choices (switch-like torque responses) to left and right in Figs. 6B–C. Changes in firing rates and LFPs in the left (red) and right (blue) optic lobes, shown when a fly chooses ipsi- and contralateral sides, respectively. (C, D) At rest (zero-torque): left stimulus boosts LFP of the left optic lobe more than right stimulus (C, bottom); the right optic lobe also prefers ipsilateral stimulation (D, bottom). Due to the one-sided stimulation of step-like movements, these differences are larger and more transient than when a fly's chooses between the stimuli (A, B). Mean firing (C, D, middle) shows less modulation as averaging cancels out ipsi/contralateral preferences of individual sites (*cf*. Fig. S8A). The data in (C, D) is from 6 flies in Fig. 4. Torque, arbitrary units; means ± SEMs shown.

Crucially, we found that neural outputs of the optic lobes showed consistent periodic activity that appeared to correlate with a fly's orientation choices over time scale of seconds (**Fig. 6A**). When a fly was choosing between the stimuli, *i.e.* generating switch-like torque responses (centre, black), LFPs (global activity) and firing of neurons (local activity) in its left (below, red) and right optic lobes (above, blue) waxed and waned, seemingly matching some slower trends in its behavior. For assisting comparisons between these responses, we use a color code in the figures. When a fly exerted torque response to right (chose the right stimulus), the activity of the optic lobes is shown on light gray background; when it exerted torque response to left (chose the left stimulus), the background is dark gray.

Because the fly's eyes were immobilized by the torque meter [24], [26], their visual input was the same. Therefore, for purely input-driven activity, adaptation within the eyes should have been equal and the outputs of the optic lobes regular and decaying over time, as happens in surgically-manipulated, fully-immobilized flies [43]. Instead, as their activity varied when the visual input did not, this modulation was not by adaptation. Nor was it caused by stimulus-related features, such as the inter-pattern (stripe) interval or spatial contrast, because the modulation in the neural outputs of the optic lobes appeared similar for different stimulus patterns (**Fig. S2D**, circles and crosses).

Three observations further strongly argued against neck or head muscle activity [44], so called clock-spikes [30], [45], [46], as the source for the modulation. First, clear action potentials could only be picked up from a small area in the left and right brain that both in resting and flying tethered *Drosophila* fired to visual motion (**Figs. 4** and **5**). Moreover, the recordings showed only little or no activity, even in flying flies, without visual motion. Second, if the electrodes were placed elsewhere in the fly brain, they typically failed to pick up action potentials both from the resting or flying flies; LFPs were then also much reduced. Finally, in these sites, firing to visual motion (**Fig. 5C**) preceded large torque responses (**Fig. 5D**). Therefore, the observed modulation in the neural activity was almost certainly generated intrinsically, either within the optic lobes or within the brain proper that links the two eyes.

### Correlating Behavior to Neural Activity

Because the fine structure of neural activity correlated weakly with the fine behavior in 1–100 ms time scales (*cf*. **Fig. 5C–D**), fast efferent flight control affected only marginally the neural responses of the optic lobes. This is not surprising, as one would not expect visual neurons to encode complex behaviors literally; particularly when visual inputs to the eyes are not affected by the behavior. Instead, their activity may reflect certain aspects of ongoing behavior. Therefore, we felt well justified to consider torque responses to left or right (over the whole duration of each response) as if these were two binary *choice states*. The activity of each optic lobe could then be time-locked by these left and right choices for comparisons.

For correlating the behavioral choices to simultaneous neural activity (**Fig. 6A**), the prolonged torque responses to left (**Fig. 6B**; dark gray background) or right (**Figs. 6C**; light gray background) were aligned by their first zero-crossings and averaged (black traces in the middle). Such estimation was reasonable as the neural activity remained vigorous throughout the selected experiments and a fly's left or right choices often lasted quite similar periods. At zero-crossings, the polarity of the torque responses flipped between left and right, having the fastest rate of change in a fly's torque response. Consequently, time-locking the responses by zero-crossings minimized jitter. The activity in the right (E#1, blue) and left optic lobes (E#2, red) was then time-locked for each behavioral choice and averaged accordingly, making their mean estimates the most reliable.

The recordings, which had many torque responses of similar time course, presented in reliable average left and right choice states. Although not prerequisite for bilateral comparisons of neural activity to binary choices, nonetheless, the first 3–4 s of the averaged torque responses often had very small SEMs. In such cases, the waveforms of a fly's left (**Fig. 6C**, downward) and right choices (**Fig. 6D**, upward) were similar but of opposite polarity. Whilst more importantly, the average neural activity, as pooled for the left or right choices, respectively, varied relatively little. That is, the corresponding outputs of the right (top, blue) and left (bottom, red) optic lobes were consistent (small SEMs) for each choice. However, their outputs differed for left or right choices. For example, compare the average LFPs and firing rates of the right optic lobe (E#1, blue traces) during left (**Fig. 6C**, E#1_L_) and right (**Fig. 6D**, E#1_R_) choices. The right optic lobe showed more activity during right choices than left ones, as its firing rate rose and LFP hyperpolarized then more. Clearly, some process was exerting its dynamics at the optic lobes in a consistent and choice-dependent manner.

### Neural Activity is Enhanced on the Chosen Side

How does this modulation affect the flow of neural information from the eyes? To answer this question, we subtracted the mean firing rates and field potentials of each optic lobe for a fly's left and right choices. In addition, the choice-dependent differences in the firing rates of local neurons were displayed as relative changes; for instance, in the left optic lobe: 100*[E#2_L_(spikes/s)-E#2_R_(spikes/s)]/E#2_R_(spikes/s). Such formulation provides an easy way to assess the relative strength of modulation on the neural output of each optic lobe.

This simple analysis exposes the powerful and dynamic nature of the modulation. In general, the activity in the left optic lobe (**Fig. 7A**, red) was enhanced (boosted) when a fly chose the left scene (black); and quite similarly, its right optic lobe (**Fig. 7B**, blue) was most active when a fly chose the right scene. During the rapid side-switching, the firing rates (centre) could increase over twofold. For some local neurons, the firing rates could in fact peak before a fly had declared its choice; before its torque responses crossed the zero mid-line (*cf*. **Fig. 7A**). Nonetheless, we found that for both left and right LFPs (bottom), the largest changes typically occurred slightly later, but still within the early phase of the behavioral choice (note, LFPs increase downwards). Significantly, the LFPs (global activity) were always enhanced on the chosen side (n = 25/25 flies), but the firing dynamics (local activity) varied with the recording sites (**Figs. 7A–B**, centre).

The inspection of the relative changes in the firing rates across all the experiments reveals a large diversity among the responses (**Fig. S10A**). We expected to see variations in the local activity from one recording site to another, because we had little control over which microcircuit each recording electrode ended up touching. As the neurons in the optic lobes are oriented retinotopically, and at the level of the lobula plates, have many cross-connections with the other eye, each receives and processes information differently [34], [35], [47]. However, our data also implies something even more fundamental about this layout. The firing patterns of neurons showed variable ipsi- or contralateral preference with variable tuning. Because of its possible evolutionary and cognitive advantages [48]–[50], dynamic signal comparisons through close arrangement of neurons, which prefer different eyes or visual aspects [51]–[54], might reflect a general wiring plan of binocular animals [55]–[57]. Thus, segregation of neurons into monocular regions within a optic lobe might advocate efficient usage of constrained neural hardware [48], [58] and improve discriminative capabilities [52], [53], [56]. Despite this potential organization, the firing of their neurons was always intrinsically modulated; when a fly chose the stimulus on a neuron's preferred side, its firing rate increased by 83±16% (mean ± SEM, n = 17) (see for example **Figs. 7A–B**, center).

Nonetheless, perhaps most remarkably, the increase in global activity in one optic lobe, when a fly chose the ipsilateral stimulus, resembled the increase in activity when this stimulus was presented alone to a resting fly (**Figs. 7C–D** **and S7B**). Thus, when a fly chose one of the two competing scenes, the intrinsic modulation made the LFPs look quite as if the fly was seeing only one scene. Naturally, in this comparison, the LFPs to the step-like one-sided stimulation (*cf*. **Fig. 4**) showed larger and more transient differences; after all, one eye did not receive any motion stimulation then. However, it is a striking finding that during competing stimulation (*cf*. **Fig. 6**) neural activity in the chosen side, as defined by torque responses, was enhanced with such comparable dynamics. Furthermore, since this modulation was somewhat similar when *Drosophila* were flying (**Figs. 7A–B**) or at rest (**Figs. 7C–D**), it was unlikely to be evoked by steering reflexes [25]; *i.e.* it should not have ascended from the haltere system in the thoracic ganglia (see Text S1).

### Intrinsic Modulation Gates the Flow of Visual Information from the Eyes

Having shown that LFPs presented a consistent global measure of how intrinsic neural activity modulated the flow of visual information in the optic lobes, we dedicate the rest of the results for analyzing these data further.

**Figure 8:**
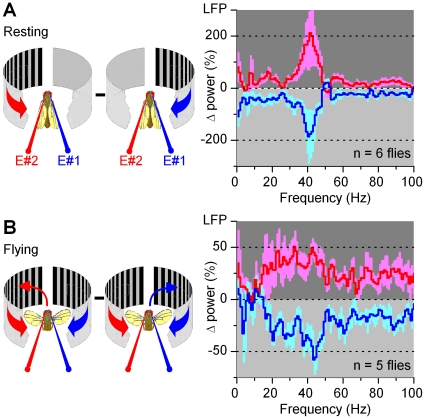
Neural activity (LFPs) increases at gamma-frequencies. Relative changes (Δ) in power spectra of neural activity in the optic lobes, when: (A) a moving screen of black and white stripes is presented to a resting fly or (B) when a fly chooses it (torque response toward it). Traces show mean ± SEM for the relative changes in LFPs pooled from experiments in different flies; E#1 and E#2 are the right and left electrodes. When presented with, or choosing, ipsilateral motion stimulus, the power spectrum of LFP in one LP increases by 20–200% between 20–100 Hz over its corresponding power spectrum for contralateral stimulus; maxima between 20–50 Hz (*i.e.* gamma-band). For details of the calculations and individual experiments, see Fig. S9.

To probe whether intrinsic modulation gates the flow of visual information from the eyes in a uniform manner, we next compared the relative changes in signaling frequencies of the LFPs when the left or right stimulus was presented to a *resting* fly (**Fig. 8A**) or when a *flying* fly chose a stimulus during visual competition (**Figs. 8B** **and S9**). The differences in the LFPs' power spectra between left or right stimulation (monocular stimulus paradigm) or a fly's left or right orientation choices (competing stimuli paradigm) were averaged for each optic lobe across different trials (see Materials and Methods). These dynamics were reproducible for individual optic lobes, but their strength and fine features varied from fly to fly (**Fig. S9**), suggesting again that the recording location influenced how activity from multitude of neural pathways was registered. Nonetheless, because the overall dynamics in each paradigm appeared sufficiently similar, we consider here the mean differences of the recordings. As expected from their bigger responses (*cf.* **Figs. 4** and **6**), the changes in the power spectra were the greatest for monocular stimulation at rest. Yet crucially, the increased activity during resting or selective orientation occurred predominantly upon similar frequencies. In both cases, neural activity increased (20–400%) in the ipsilateral optic lobe at 10–100 Hz; peaking at 20–50 Hz, at gamma-band. This band of frequencies has been reported to signal attention like processes in *Drosophila* [14], [59] and is often associated with synchronized oscillations of synaptic networks and cognitive processes in higher animals [60], [61].

**Figure 9:**
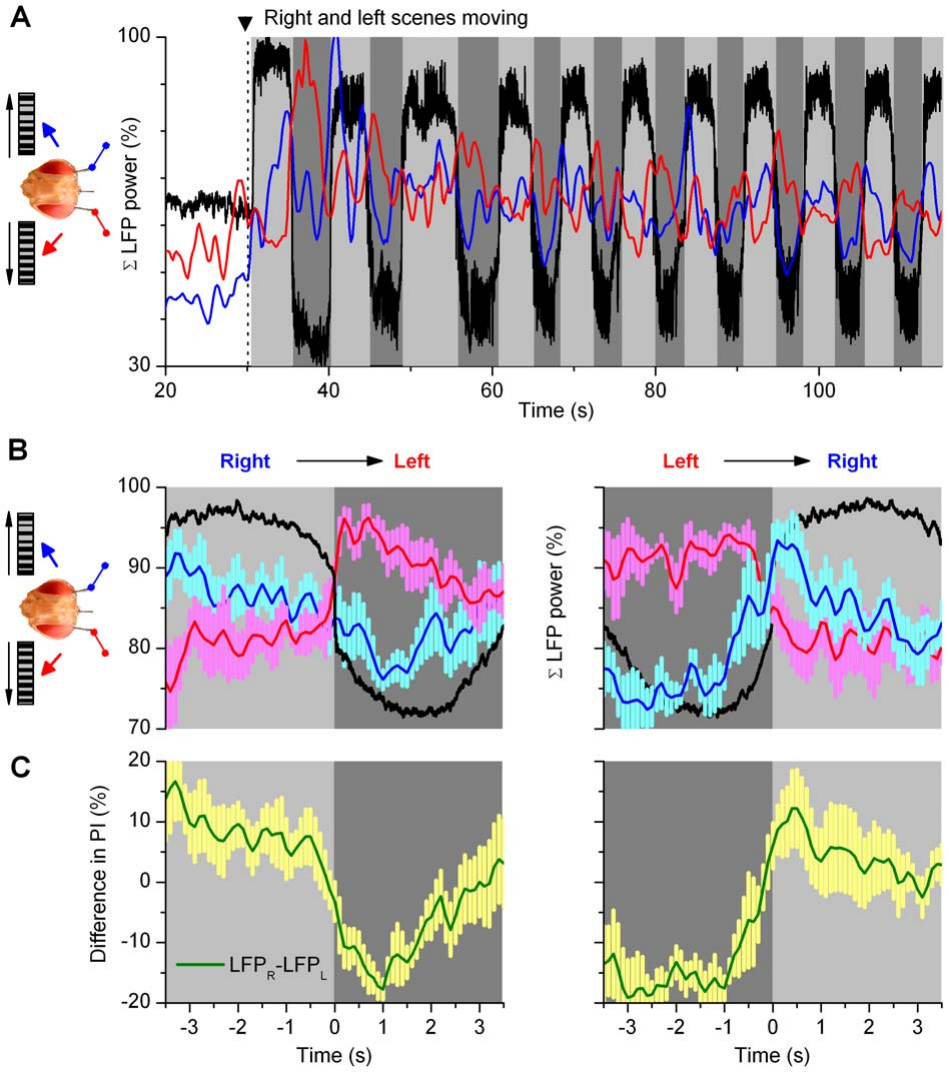
*Drosophila* brain gates the flow of visual information from the eyes. (A) A fly faces identical screens of black and white stripes on its left and right, and we measure the outputs of its left (red) and right (blue) optic lobes, as power-indexes (20–100 Hz frequencies) of their LFPs. When the scenes are set to motion, the left and right power indexes oppose each other (*i.e*. these are 180° phase shifted), alternating in synchrony with the orienting behavior (black). Light grey sections highlight switch-like torque responses to right; dark grey sections to left. Notice also the effect of saliency in the power index; the overall neural activity settles down from the initial maxima as the fly continues choosing between the stimuli. (B) The behavior-triggered average of the right (blue) and left (red) optic lobe's power-indexes during right-to-left and left-to-right torque responses (black); torque, arbitrary units. When a fly's orienting flips sides, its brain activity is readily enhanced on the chosen side but more gradually suppressed on the opposite side. (C) The difference in power-indexes (green) predicts the behavior in (B). Mean ± SEM of 5 flies. For details of the calculations and individual experiments, see Fig. S10.

Such modulation could result from a neural mechanism, which excited one optic lobe or, in addition, inhibited the opposite. Here the modulation appeared excitation-inhibition-coupled, as identified by calculating a continuous power-index for the relevant 20–100 Hz band of neural activity (* = * filtered variance in the time domain) in the left or right optic lobe (**Figs. 9A**; red and blue traces, respectively). When normalized, these simple metrics - for tracking the bilateral neural outputs over time - make it easy to see how the activity of the optic lobes changed during an experiment. Although modulated in synchrony with the fly's orientation choices, *i.e.* torque responses (black), the left (red) and right (blue) indexes mostly opposed each other (180° phase shift). Thus, when a fly chose one stimulus, the optic lobe on this side was excited and the opposite optic lobe was inhibited. Hence, intrinsic modulation - either from the higher brain centers or within the optic lobes or from both - gated the flow of visual information from the left and right eyes in a coordinated manner. In a purely mechanistic view, the modulation increased activity in the attended side and decreased activity in the ignored side. Interestingly, the LFPs also showed signs of stimulus saliency [59]; as the scenes started to move, the indexes could jump up ∼50% before stabilizing to a lower baseline.

To reveal the relative strength and time-course for these opposing signals in the optic lobes, we further calculated behavior-triggered averages of the power-indexes when the fly's orientation choices of stimuli shifted from right-to-left or left-to-right (**Fig. 9B** **and S10**). Because the average power-indexes (**Fig. 9B**) of left and right optic lobes (n = 5 flies) are similar to each other and to those of individual optic lobes (**Figs. S10**), these findings make a powerful case that (**1**) gamma-band changes in the LFPs, irrespective of their exact recording location, are real, reproducible and general, and that (**2**) the neural output of each optic lobe is modulated with comparable 2-phase dynamics during selective orientation. The excitatory modulation to optic lobes (left, red; right, blue) seemed in part anticipatory, as the indexes typically rose before the flies settled pursuing the ipsilateral stimuli, peaking at the times (or before) the behavioral choices (black) switched sides, whereas the inhibition was weaker and slower (left, blue; right, red). Thus, the distinctive but coupled excitatory and inhibitory modulations make it unlikely that the left and right eye were rhythmically inhibiting each other and that the motor system was simply steering to the side where from most information flows. Instead, this modulation requires further processing stages.

Finally, we note that by subtracting the low-frequency components of the opposing optic lobes (*i.e.* balancing excitatory and inhibitory loads; **Fig. 9C**) a post-synaptic mechanism could predict the fly's choices (**Fig. 9B**, black) reasonably well.

## Discussion

We investigated the role of intrinsic brain activity in visual information processing in *Drosophila* that faced two competing stimuli (monocular flow fields) in a customized flight arena (**Fig. 1**). A tethered flying fly exerted switch-like torque responses between the stimuli, as if it saw only one stimulus flipping side-to-side. Thus, it reacted by choosing one stimulus at a time (stimulus selection) (**Fig. 2**). This interpretation is strengthened by the observations that it could also fly straight, or even against a unilateral stimulus, which would be impossible if its torque responses were simple optomotor steering reflexes. Furthermore, in repeated trials, a fly took a highly variable time of hundreds of ms to make its first orientation choice (**Figs. 3A, C**), which often varied haphazardly between the stimuli (**Fig. 3B**). Such great variability makes this behavior very different from the classic optomotor steering reflexes, which are more tightly phases-locked to optic flow. By using miniaturized electrodes lodged in a fly's left and right optic lobes (**Fig. 4**), we explored how their neural activity correlated with the visual stimuli and with the fly's orientation choices (**Figs. 5**, **6**, **7**, **8**, **9**). We first showed that neural activity was predominantly stimulus-driven and that it always preceded the behavior (**Fig. 5**). Through using both monocular stimulus and competing stimuli paradigms for resting and flying flies, we then identified additional periodic activity, which was neither set off by the visual stimuli nor a recording artifact, but occurred when a fly was making choices (**Figs. 6** **–** **7**). This modulation, which is likely to arise from circuits' internal dynamics, resulted in a gating-process that enhanced the overall output (LFP) of the optic lobe facing the chosen stimulus while the output of the opposite side was suppressed (**Figs. 8** **–** **9**). The difference between these signals, distributed around gamma-frequencies (20–100 Hz), could in part predict a fly's orienting behavior (**Fig. 9C**).

Together these results imply that when a fly decides to turn (for whatever cause), intrinsic activity acts upon the optic lobes to modulate visual input from the eyes. Interestingly, the output of this modulation, whether intentional or due to noise, resembles top-down sensitivity control [4], [62]–[65].

This study further revealed a somewhat surprising strength of reciprocal interactions between the opposing eyes on responses of individual neurons (**Figs. 5** **–** **6** and **S8**). While there has been a variety of electrophysiological approaches for studying signaling in the visual pathways of flies in various passive preparations, *e.g.* [66], [67], our study is perhaps one of the first ones to simultaneously look at neural responses to visual motion at the left and right optic lobes during active behavior (but see [68]–[71]). As was shown in the cricket auditory system that uses corollary discharge to reduce the effect of self-generated signals [3], and recently in *Drosophila* lobula plate [12], [13], analysis of sensory pathways in passive preparations does not provide a full view of their dynamics in active animals [10].

### Neural Mechanisms of Intrinsic Modulation?

The experimental techniques we used for this study cannot establish with certainty the neural origin of the intrinsic modulation. Nonetheless, it is safe to say that the variable dynamics for excitation in the optic lobe of the chosen side, and for inhibition in the opposite side, suggest interlinked chains of events between peripheral processing and central initiation of actions. The activity in these circuits could be noise-induced, intentional or both.

Imagine a toy-model for unstable flight motor equilibrium. Two neurons (L and R) receive visual inputs, integrating slowly. Both neurons are noisy and have variable thresholds. If L's threshold is reached first, a left turn is triggered; similarly, crossing R's threshold triggers a right turn. A turn then offsets both neurons, resets their thresholds and the integration starts again. Although such simple circuits cannot generate and couple *faster increase* in the ipsilateral output to that of *slower decrease* in the contralateral output, before triggering the choices (**Fig. 9**), one can imagine more advanced cross-brain connections when coupled to a noisy motor pattern generator, which could do this. Thus, while we cannot disprove the role of noise in our findings, it seems reasonable to expect sophisticated neural interactions, which involve the central brain [6], [8], [72], [73] for the flies' orienting behavior.

Single neurons in the auditory system of crickets provide corollary discharge information (efference copy) to maintain auditory functions, while generating loud courtship songs during vocalization [2], [3]. Could, similarly, gating of visual information by intrinsic modulation reflect efference copy of the orienting behavior?

When a fly orients toward a stimulus, such efference copy could be used to predict the location of the expected visual input in respect to its eyes. In such scheme, the expected outcomes of the orienting would be fed to the neural circuits of the optic lobes that encode visual motion inputs. This efference copy would then converge with real visual inputs resulting from the ongoing orienting behavior. The difference or deviations between the expected and real visual inputs would be used to rectify the fly's orienting. While the fly brain perhaps sends a real-time copy of the initiated motion innervation to the optic lobes, our data gives no clear evidence for this proposition. The main intrinsic modulation seems too slow; it occurs in a prolonged time scale (*cf.* **Fig. 9**). The fast transient spike patterns may in part reflect correction signals (for instance some exploratory saccades show correlated activity; **Fig. 6A**, stars) but the general intrinsic biphasic modulation of the opposing optic lobes works more likely to enhance the fly's discriminative capacities.

Such modulation could be attributed to rivalry (crossing signals from the optic lobes interact with the brain centers between) or to more central top-down activity (descending inputs from the higher brain centers). For conventional binocular rivalry, both the left and right eye view different objects but their information is processed using the same overlapping visual field (stereopsis) [65]. In our paradigm, this seems unlikely. As the left and right eye of the fly saw separate scenes with visual fields that do not overlap (monocular flow fields), their perception should be stable. Nonetheless, the fly brain may spontaneously generate rivalry between its left and right optic lobes by interhemispheric switching of their activity states, as has been detected in higher animals, including humans [74]–[76]. Since *Drosophila* can also fly straight (**Figs. S3** and **S5–S6**), and during unilateral stimulation even turn to the opposite way (**Figs. S7**; see also [16]), these results imply that it is likely to possess some form of voluntary control over its orientation in the tested behavioral paradigms. Therefore, in light of the recent findings about the activity-dependent gain-control of lobula plate tangential neurons [12], [13], our results make a reasonable case that when a fly moves it activates attention-like circuits to better discriminate (or partition) relevant changes in the optic flow in respect to its orientation choices (turns).

### Limitations of This Study and Possible Supportive Evidence

We recognize the many limitations of our study: the locations of the miniature electrodes are not known at the level of circuitry; the sensitivities of the electrodes can vary between preparations; perhaps our competing stimulus paradigm is too simple to test attentive processing, or too similar to the classic optomotor steering paradigm; the open-loop setting may stress the flies; there is a possibility of noise pollution from the muscle activity, and so on. Therefore this study is far from being conclusive, and many of its results and ideas need to be further tested by other methods for verification or disproval.

**Figure 10:**
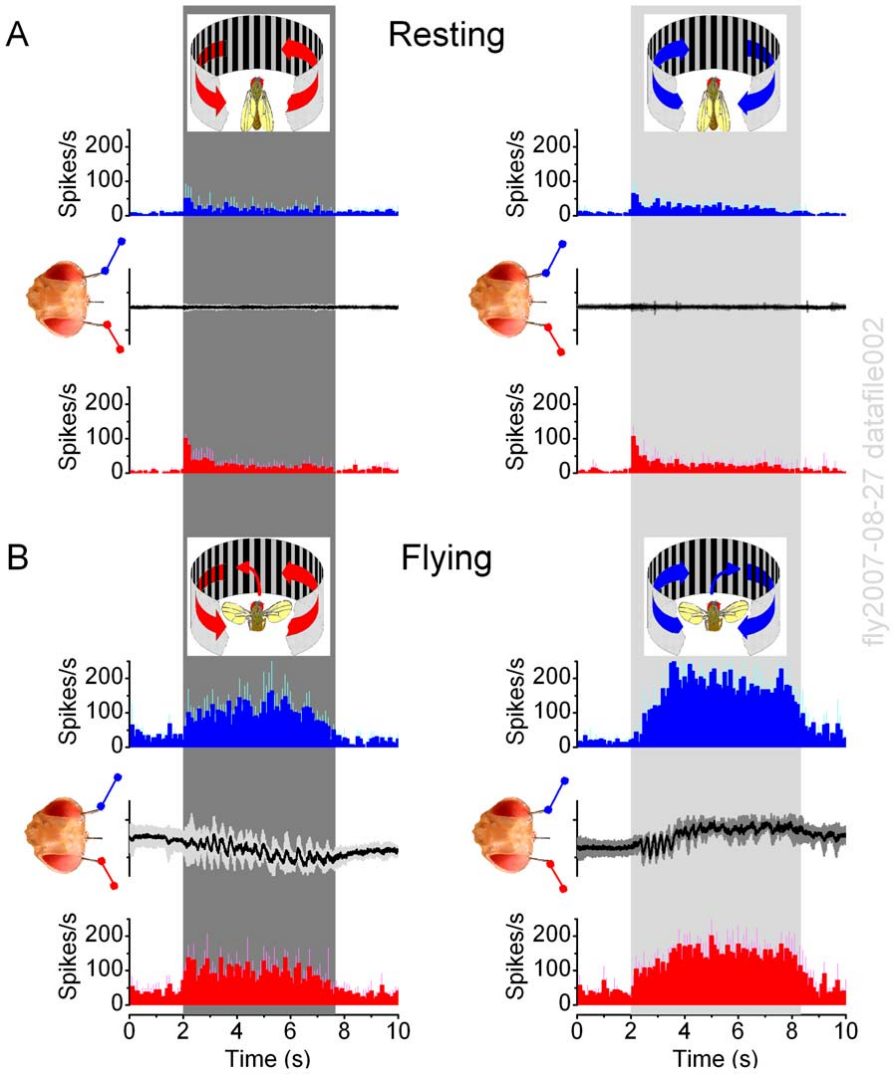
Neural activity in the optic lobes, as measured by our miniature electrodes, depends on the fly's behavioral state. (A) Firing activity in the left (red) and right (blue) optic lobes of a resting fly to leftward and rightward field rotation. (B) Neural firing in the optic lobes of the same fly, but when flying, to leftward and rightward field rotation, which initiates corresponding optomotor responses (black). Neural activity in the optic lobes can increase 2-3-fold when flying. (Mean ± SD, n = 5 trials in each experiment). Torque in arbitrary units.

Interestingly, however, recent experimental results from patch-clamping visually identified lobula plate neurons in *Drosophila* [13] and detecting their calcium changes [12] may give new support, at least, for the reliability of our findings. Flying or walking amplifies their neural activity to moving stimuli well above what is seen when the flies rest (increasing their output by 2-3-fold). Since we regularly observed similar activity dependency between the resting and flying states (see for example **Fig. 10**), it is plausible that our miniature microelectrodes could equally detect the gating of the optic lobe gain, as we suggest in this paper.

## Materials and Methods

### Flies

Wild-type *Drosophila melanogaster* (Canton-S) were raised on standard medium at 22°C and 60% relative humidity under a 12/12-h light/dark cycle. Three to four day-old females were immobilized by cooling (<3 min) and small copper-wire harnesses (hooks) were glued between their head and thorax, using UV-sensitive glue (Loctite). Flies then rested overnight in single vials having sucrose and water.

### Flight Simulator System and Behavioral Experiments

A tethered fly was connected to the torque-meter by a small clamp holding the copper-wire harness. Suspended between two taut wires, which acted as torsion springs, and damped by magnets, the torque meter's centre-axis supports a miniature mirror that reflects changes in the yaw torque of the flying fly (**Fig. 1A**). By pointing a laser-beam to the mirror, its light-return over distance amplifies the yaw torque signal, which was then transduced to voltage by an optical sensor. The measured light-return was calibrated and found to be linear with respect to applied torque. The system has a fast rise-time and high signal-to-noise ratio (**Fig. S1**).

At the torque-meter, a *Drosophila* was fixed in a rigid position and orientation, flying stationarily [24], [26]. Here its eyes/head could only move <0.03°. Because this is <1/160^th^ of the inter-ommatidial angle (∼5°) that defines its eyes' spatial resolution [77], [78], the fly's body movements were not expected to affect the stream of images it saw during the experiments.

Perpendicular to the fly's long axis, facing its left and right eyes, were two semi-circular screens presenting competing visual stimuli. They displayed printed patterns (stripes, crosses or circles) on two identical paper-strips. The strips were spun by a stepping motor, generating two equal scenes that swept to the opposite directions (left and right) synchronously. This simple mechanism made the scenes continuous; it was free of artificial motion, flashing and aliasing. Typical stimulus parameters for moving stripe scenes were: azimuth ±150°; elevation ±40°; wavelength, 20°; velocity, 60°/s; contrast, 1.0, as seen by the fly. These values represent the maxima (or minima) also for the crosses and circles (**Fig. S2D**) that were smaller and more separated. The scenes were illuminated by day-light and/or by a cold-light-source via fiber optics.

In a competing stimuli paradigm, a tethered flying fly, which is heading straight (zero-torque), is suddenly presented with two motion stimuli (monocular flow fields) of equal strength (**Fig. 1B**), one on its left and the other on its right. After a neural processing delay, a fly intends to turn either to left or right, as seen by yaw torque (**Video S1**). This orientation response is taken as a fly's *report* for the chosen stimulus, whereas two microelectrodes, implanted in its left and right optic lobes (below), are used to look for neural signatures (in multiunit action potentials and local field potentials) for this choice. We call the resulting 3–20 s long, side-to-side slipping, square-waved responses simply as torque responses to distinguish them from the classical optomotor responses to continuous field rotation that are smaller and much briefer (**Fig. 2D**). Importantly for purposes of analysis (see below), each orientation choice was considered a binary state (or choice state), which lasted the period of the torque response. For example, a *left choice* started when a torque signal crossed the zero-midline to left, and it ended when the signal crossed the zero-midline again (to point right).

The behavioral results of this article were further confirmed by additional experiments in which the competing motion stimuli were delivered via fiber optic bundles on the two hemifields of a cylindrical arena that surrounded the tethered flying fly. The arena contained a dense grid of 128×4 optical slits (pixels), covering 360°; thus, each slit extended horizontally 2.81°, as seen by the fly. Light output from clusters of LEDs were channeled into columns of slits under user-control, generating moving stripe patterns, whose speed, intensity and horizontal width could be altered during the experiments. The competing motion scenes in both systems were efficient in evoking torque responses of similar general dynamics, as tested by different stimulus patterns, speeds (*cf*. **Fig. S5**) and luminances (Text S1), making this paradigm robust.

### Electrodes

We designed a miniature electrode with a soft connecting wire (**Fig. S2A**) that left the fly's visual behavior and torque measurements undisturbed. 20 µm (Ø) tungsten rods were thinned by gravitational pull and current injection before cutting them into 1 cm sections. A small (Ø 20 µm) insulated copper wire was welded to each rod 1.5 mm from its tip. The rods were sharpened with standard electrolytic procedures to taper 30°, insulated by polyimide resins (leaving the finest 30–50 µm tip exposed), and cut to 1.5 mm lengths with the wires at their end. Their impedance varied 1–1.5 MΩ.

Three miniature electrodes – the left, right and reference - were glued to the small clamp that attached the fly to the torque-meter (**Fig. 5A**). The fly was clamped and the electrodes were inserted by hand (**Video S2**) in the chosen brain areas in about 100–150 µm depth. The electrodes were wired to a connector-block, taking their signals via shielded cables to the high-impedance amplifiers (Cerebus-128, Cyberkinetics, USA).

In trials, we inserted up to six electrodes in the brain for finding the best location to record neural activity to visual moving stimuli. The recording sites with sufficient signal-to-noise ratios were rare and the rate of successful experiments was low. We tested micromanipulators to place the miniature electrodes, but given the small dimensions about the set-up, manual insertion under stereomicroscope was deemed to be the most efficient technique. Eventually, we learned that LFPs and action potentials could be picked up reliably when the electrodes resided at the distal region near the dorsal eye rim; where the last neuropile of the optic lobe, the lobula plate (http://flybrain.neurobio.arizona.edu/), is located. Placing an electrode in each of these sites, about a centrally positioned reference electrode, could give electrophysiological data for hours. Based on their motion-selectivity and rapid adaptation (**Fig. 4**), typical for large tangential cells (LPTCs) [43], we concluded that the electrodes probably were in the lobula plates. These neurons were insensitive to light intensity, sound or mechanical stimulation. Back-to-front motion also increased their activity, but since it evoked weaker and less clear torque responses (**Fig. S4**), this stimulation was not studied further.

During the experiments, the flies were monitored to ensure that their responses were not induced by, or related to, spurious muscle activity or self-induced visual motion stimuli, *i.e.* rubbing the eyes or lifting up the proboscis to the visual field. Although such activity can disrupt LFPs and spike rate measurements, being quite common with some resting flies, we did not see this with flying flies; the flies typically flew with their legs neatly dangling under the abdomen, even during switch-like torque responses (**Fig. 5A**; **Video S3**). For the resting flies, we eliminated the data sections in which the fly was active “grooming and trumpeting” from the analysis, such as for **Figure 4**. However, when flying, considering the hours of recordings from successful experiments, even if there were few such events, these could affect the results only little. More details are in Text S1.

### Data Analysis

Signals were processed by a Cerebus-128 system (Cyberkinetics, USA). The spikes were amplified 5,000-fold; high-pass filtered at 0.5 kHz, low-pass filtered at 7.5 kHz; sampled at 30 kHz with 16-bit resolution. The LFPs were low-pass filtered at 0.25 kHz and high-pass filtered 0.3 Hz. Together with yaw torque and speed of the moving screen, LFPs and spikes were sampled at 1 kHz, monitored on-line and stored in a hard-drive. The spikes were detected using a discriminative threshold; with a spike-sorting algorithm counting each spike only once (**Figs. S2B–C**). Their waveforms and patterns, and other signals were analyzed using custom-written software [40], [79].

There are three important points to consider when searching for correlations between neural activity of the left or right optic lobes and the fly's orientation choices:

1. Because of the sensitivity differences and variable recording locations of individual electrodes, one should not directly compare their unprocessed signals. However, for the same experiment, activity of each optic lobe can be analyzed separately. For example, one can compare the signals in the left electrode for left and right choices, given that its sensitivity does not deteriorate and the orientation choices are clearly distinguishable.
2. Because highly variable single trial behavior (*cf*. **Fig. 3**) correlates weakly with the fast neural activity of the optic lobes (*cf*. **Fig. 5**), one is justified to search for slower associations by linking neural activity to the binary *choice states*, even though the fine structure of torque responses vary. Slow associations, as signs of intrinsic modulation, can be quantified consistently in a prolonged trial, where a fly's has made many left and right choices, by averaging the neural responses during all left or right choices of similar duration.
3. Once neural activity picked up by an electrode is analyzed for the choices, results from different trials or flies can be compared for similar trends. If such trends seem frequent, their generality can be then established by pooling the representative results from different experiments; given that in each case the recorded neural activity occurred during similarly patterned orientation choices.

Therefore, the intrinsic modulation (Δ) of one optic lobe (or electrode) was estimated from the torque-trigged averages of the electrical activity (LFP or firing rate), as a difference between left and right orientation choices. For the right lobe: E#1 (*right*) – E#1 (*left*) (**Fig. 7B**); in the left lobe: E#2 (*left*) – E#2 (*right*) (**Fig. 7A**). E#1 and E#2 are the right and left electrodes, respectively, as in **Fig. 5A**; *left* and *right* are respective torque responses (or choices: **Figs. 7A–B**) (moving scenes: **Figs. 7C–D**). In some of the plots, the intrinsic modulation was given as a relative changes in a percentage scale: 100*(E#2 (*left*) – E#2 (*right*))/E#2 (*right*) or 100*(E#1 (*right*) – E#1 (*left*))/E#2 (*left*), to highlight its dynamic strength changes (firing rates in **Figs. 7A–B**; power spectra of LFPs in **Figs. 8A–B**).

LFPs of the selected data sections were segmented into 50% overlapping stretches (1,000 points) and windowed with a Blackman-Harris 4^th^-term window [80] before their spectra, LFP(f) were calculated with an FFT algorithm. The spectra were then averaged to improve the estimate. For power spectrum, 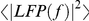, || denotes the norm and 〈 〉 the average over the different stretches.

For an optic lobe's power-index, we calculated the power spectra of its LFP using 1,000 point window, moved in 100 or 200-point steps (**Figs. 9B and 9A**, respectively). From each power spectrum a 20–100 Hz range was summed, giving us a continuous account of the dynamic changes in these frequencies at 100 or 200 ms time-resolution. For fair comparison of the activity in the left and right optic lobes, their power-indexes were normalized by maxima, and given in a percentage scale (**Figs. 9A–B**).

To emphasize the mean trends of the LFP power-indexes for both optic lobes (**Fig. 9A**), these signals were smoothed with Savizky-Golay 2^nd^-order function using 5 data points. This procedure eliminated extraneous “noise”, attributable to the many data points used for each trace, but as executed, this had little effect on the shape and timing of the mean trends. No smoothing was used for **Figs. 9B and C**, which have twice as high data-point density than **Fig. 9A**, as averaging data from 5 flies smoothed the traces naturally. More details are in Text S1.

## Supporting Information

Text S1 (0.12 MB DOC)

Figure S1 Output of the torque meter to an electro-magnetic pulse (input) shows a fast rise-time and signal-to-noise ratio. The rise time is 12.5 ms (red dotted line).
(0.08 MB TIF)

Figure S2 Spike detection, and orientation with different competing stimuli. (A) The standardized positions for the recording electrodes in the head of a flying *Drosophila*. The small harness, glued between the head and body (the thicker silver wires), was used to clamp the fly's head in a fixed position and orientation. The miniaturized electrodes are the black thin wires. Electrical responses from a fly's optic lobe (most likely from the lobula plate) to a single moving field, recorded with a miniaturized tungsten electrode. (B) Continuous voltage signals (blue) during a constant velocity motion (stripes) stimulus on the right scene (light grey, below) recorded from the right optic lobe of a resting (non-flying) fly. The spikes are detected by a threshold (red dotted line). (C) Mean spike (blue) and individual spikes (light cyan). (D) Characteristic behavior (yaw torque, black) and neural responses in the optic lobes (firing rates, above; LFPs, below) to o-patterns that move to opposing directions at 60°/s; recorded form tethered flying *Drosophila*. During the experiments both eyes receive continuous motion stimuli, as indicated by the fly-head cartoon. Similar to stripe-patterns (cf. Fig. 6), the fly attempts to follow either a left or right moving scene at any one time (i.e. generating torque responses to left or right); the neural output of its optic lobes (right, blue; left, red) are tuned with these responses. Notice the variable time courses of the left and right torque responses of this fly; compare the behavior of five other flies in Fig. 6 and S3.
(5.70 MB TIF)

Figure S3 In competing stimuli paradigm, the dynamics of the switch-like visual selection vary from fly to fly. The figure shows the orienting behavior of four different flies during bilateral visual motion at 60°/s. Before the motion stimuli, the tethered flying flies can generate exploratory saccades to left and right (stars). When the scenes move, the flies begin to generate larger side-to-side flipping torque responses toward the left (down) or right stimulus (up), one at a time. Intermixed with these responses, some flies also orient straight for brief periods (gray arrows). (A) A fly with a 3-state orientation responses; these mostly flipped between the left and right scenes, but it also flew straight frequently. Interestingly, from the stimulus onset, this *Drosophila* flew straight for about 4 seconds (apart from few saccades to right) before first choosing the left stimulus. (B) A fly that generated rather symmetrical torque responses between the left and right scenes with a slight preference to the left scene. (C) A fly whose torque responses transiently flipped between the left and the right scenes, but it oriented mostly straight. (D) A fly that generated symmetrical torque responses between the scenes, but chose to orient mostly toward the right scene. The insets show the corresponding probability density functions.
(1.34 MB TIF)

Figure S4 In competing stimuli paradigm, a fly's switch-like orienting between the left or right stimuli is evoked efficiently by front-to-back motion. The figure shows traces of behavior (yaw torque) of the same tethered flying *Drosophila* to (A) front-to-back and (B) back-to-front motion (bilateral stripe scenes: azimuth ±150°, elevation ±40°, velocity 60°/s). When the scenes are moving (light gray areas), the fly begins to exert torque responses between the left (down) and right scenes (up). These responses are much stronger for the front-to-back than for the back-to-front motion.
(3.46 MB TIF)

Figure S5 In competing stimuli paradigm, a fly's switch-like orienting occurs similarly for different sizes of motion scenes (cf. Fig. S6). The figure shows the behavior of the same tethered flying *Drosophila* to smaller and bigger stimuli (gray sectors in the circular inserts): (A) at 54° lateral scenes (126° in the front and back blacked out), and (B) 83° (69° blacked out frontally and 125° dorsally), respectively. In both cases, when the scenes are set in motion (60°/s; dotted line), there is a variable delay before the fly starts to generate side-to-side flipping torque responses, attempting to rotate toward the left (down) or right stimulus (up). Intermixed with these responses are periods of flying straight (approximately zero torque; green arrow heads), implying that the fly can also oppose the stimuli. Here, the field size of stimulation seems to have little influence on the size of torque responses. These results imply that torque responses are not evoked by some specific features in the stimulus patterns, but that they define the fly's choices. Insets show the corresponding probability density functions.
(0.73 MB TIF)

Figure S6 Visual selection occurs switch-like at different speeds of competing stimuli (150° left and right). The figure shows the orienting of the same tethered flying *Drosophila* during competing visual stimuli paradigm at different speeds: (A) at 15°/s, (B) 30°/s, (C) 60°/s, and (D) at 120°/s. Before the motion stimuli, the fly makes characteristic exploratory saccades to left and right (stars). When the scenes are set in motion, the fly generates larger side-to-side flipping torque responses to the left (down) or right stimulus (up), but it also can orient straight (small gray arrows). Although increasing the speed of stimulation appears to amplify the torque responses (cf. A and D), this evokes no obvious/trivial causal changes on their duration and frequency. These results imply that the switch-like orienting is not heavily dependent on the stimulus speed. Insets show the corresponding probability density functions.
(1.93 MB TIF)

Figure S7 A tethered flying *Drosophila* can react against unilateral motion field. Although the right scene, R, moves, the fly exert its torque response to the opposite, unmoving side (the blank left scene, L). (A) Even at the onset of the unilateral motion, a fly may occasionally respond against it by generating a torque response toward the opposing blank scene. (B) Also when engaged in pursuing the unilateral motion, a fly sometimes briefly interrupts its response to orient toward the unmoving blank scene. This unexpected behavior highlights that even if its reactions are triggered by a strong moving field-stimulus, these can be modified at any one time by internal motor commands.
(2.85 MB TIF)

Figure S8 Differences in local and global neural activity. Firing patterns of individual neurons (A) in the optic lobes show variable tuning for ipsi- (blue traces) and contralateral (red traces) visual motion, (B) but the global activity of the optic lobes (LFPs) always increases when selecting (torque responses toward) ipsilateral scenes. Except for torque (C), traces show the relative change in responses toward the preferred side; aligned by the torque responses. By their preference for ipsi- or contralateral stimuli, the neurons in the LPs can be sorted into four groups (data from 9 flies, from 17 neurons [thus from 17 different electrodes]; bin-size 100 ms). *Transient increase* neurons fire the most at the early part of the fly's torque response to ipsilateral objects. *Slow increase* neurons steadily increase their firing rate, until the ipsilateral torque response starts to ease off. *Transient decrease* neurons respond best at the beginning of a shift to contralateral side. *Slow decrease* neurons are most active when the torque response toward the contralateral stimulus peak. Unlike the firing rates, the absolute amplitudes of the corresponding LFPs always reach their maxima (i.e. points downwards) during ipsilateral viewing. Means ± SEMs shown.
(0.30 MB TIF)

Figure S9 Ipsilateral neural activity is enhanced during visual choice paradigm. This figure shows typical changes in the power spectra of LFPs, as measured from the optic lobes during left and right torque responses of a single fly. (A) The fly generated 14 switch-like torque responses to left and right during bilateral motion stimulus (stripe-bars). Mean torque responses (black) to left (down) and right (up) are aligned with the corresponding mean LFPs, measured by the right (E#1, blue) and left electrode (E#2, red). Scale: 2 s/300 µV. Gray areas indicate the sections across LFP recordings that were used for calculating the power spectral estimates. (B) Power spectral estimates (mean ± SEM, n = 98 samples) for the left optic lobe (left) and the right optic lobe (right) during left (red and navy LFPs) and right (blue and wine LFPs) choices. (C) Relative differences in the neural activity in the left (red) and right (blue) optic lobes during the left and right choices (mean ± SEM, n = 14 torque responses). Similar to the pooled data across the flies (Fig. 8B), here the ipsilateral signals are boosted at ∼20–90 Hz.
(2.85 MB TIF)

Figure S10 Intrinsic modulation of neural activity in the optic lobes during the competing stimuli paradigm; visual motion input to the eyes remains unchanged, yet the neural outputs of the optic lobes oppose each other. Changes in the mean neural output of the left and right optic lobes (OL), monitored as the summed power (20–100 Hz) of their LFPs, coincide with the torque responses. (A) LFPs from the right (blue) and left (red) optic lobes; yaw torque (black) shows attempts to rotate to right (up) and left (down), highlighted by light-gray and gray bars, respectively; data from the same fly as in Fig. S9, again with 14 torque responses to left and right. (B–C) shows neural activity during right-to-left and left-to-right choices. 14 LFPs from the right (cyan) and left (magenta) optic lobes superimposed and aligned by the corresponding zero-crossings in the torque (yellow). The means are shown in blue, red and black, respectively. (D–E) Power indexes of LFPs are then calculated for each separate torque response; here, giving 14 power-indexes for left and 14 power-indexes for right choices. For, each torque response we used a 1,000-point sliding window with 100-point jumps (B) and their frequencies from 20–100 Hz are summed up for each data-point. The mean ± SEM of these 14 power-indexes are shown in (D) and (E) for right-to-left and left-to-right choices, respectively. Changes in the output of the left and right optic lobes precede the changes in the torque responses, oscillating with a 180o phase shift. (F–G) shows similar dynamics in the power-indexes of another experiment (Fig. 6). (F: mean ± SEM). These general dynamics in LFPs are also seen in the pooled data for five flies, as shown in Fig. 9B.
(9.84 MB TIF)

Video S1 This video shows torque responses of a tethered flying fly flipping between the right and left competing motion scenes.
(1.24 MB MOV)

Video S2 This video shows how the miniature electrodes were placed manually into the optic lobes of a tethered flying Drosophila.
(0.59 MB MOV)

Video S3 This video shows a flying tethered Drosophila with three miniature electrodes firmly placed in its brain.
(1.13 MB MOV)
