# Supplementary material for: Intrinsic Activity in the Fly Brain Gates Visual Information during Behavioral Choices
Source: PLoS One. 2010 Dec 30;5(12):e14455. doi: 10.1371/journal.pone.0014455 (PMC3012687; doi:10.1371/journal.pone.0014455)
Supplement: Text S1 — (0.12 MB DOC) [file pone.0014455.s001.doc]

**Intrinsic Activity in the Fly Brain Gates Visual Information during Behavioral Choices**

Shiming Tang^1,2^ and Mikko Juusola^1,3^

*^1^State Key Laboratory of Cognitive Neuroscience and Learning, Beijing Normal University, Beijing 100875, China*

*^2^State Key Laboratory of Brain and Cognitive Science, Institute of Biophysics, Chinese Academy of Sciences, 15 Datun Road, Chaoyang District, Beijing 100101, China*

*^3^Department of Biomedical Science, University of Sheffield, Sheffield S10 2TN, UK*

**CONTENTS** Page:

**I. Using the Modified Flight Simulator System** 1

**II. Using Miniature Electrodes** 2

**III. CONSIDERING DIFFERENT VIEWS** 3

**III.1. unstable flight motor equilibrium** 3

***I.1.1. Steering-reflex hypothesis: Behavior*** 3

***I.1.1.1. Behavioral evidence challenging the steering-reflex hypothesis***. 4

**IV. NEURAL ACTIVITY** 5

**IV.1. Differences between Firing patterns and LFPs** 5

**IV.2. Changes in power spectra of LFPs during opposite torque responses** 6

**V. Could intrinsic modulation arise from the thoracic ganglia?** 7

**VI. REFERENCES** 7

**I. Using the Modified Flight Simulator System**

It was fundamental for our experimental aims to have a device that could reliably and repeatedly present visual stimuli to a fly while measuring its behavioral responses. Here, an adaptation of the *Drosophila* flight simulator system served this purpose, as it has for many other vision related questions [1-13]. The adapted design (**Fig. 1A**) reflected the need to present alike competing stimuli to the left and right eyes while measuring neural responses of the optic lobes in low noise conditions. Whilst these alterations were suited for our specific experiments, the general concept of measuring yaw torque as a fly’s behavioral response to visual stimuli did not change. Thus, the large square-waved torque responses of flies to competing stimuli (**Fig. 2**) are not anomalies of the optical torque-meter used in this study, but they look similar in the classic electromagnetic torque-meter. The optical torque-meter (Materials and Methods) was used because of its smaller size and superior signal-to-noise ratio, frequency range and electrical noise properties (*cf*. **Fig. S1**) that made the simultaneous electrophysiological experiments feasible.

In the context of the behavioral assay, we would like to emphasize two important points about the results. First, whilst activity differences between the left and right optic lobes clearly seem to be established intrinsically, besides them *reflecting* different behavioral choices*,* we make no claim that this visual imbalance would *directly* cause the fly’s torque responses. Presumably, the underlying neural mechanisms are more sophisticated, and may involve intricately coordinated parallel processing between multiple sites, similar to species with bigger brains [14]. Second, there remains some degree of ambiguity in the interpretation of flies choosing between the left and right stimuli by “attraction”. For example, a fly takes variable time to exhibit switch-like torque responses, but once it starts, it only sporadically flies straight again (**Fig. S5**). Intuitively, its excessive side-to-side flipping may seem reflex-like. Moreover, as a fly tries to turn toward one side and cannot succeed because its head is fixed, it might experience “stress”, which could influence the results. Because the competitive stimuli paradigm, by its design, quantifies only choices, not their causes, the exact levels of voluntariness and willingness of these behaviors remain debatable.

**II. Using Miniature Electrodes**

In larger animals, with bigger brains, field potentials recorded with sharp tungsten needles are truly local. However, in the small *Drosophila* brain, the field potentials seem more global than local. Thus, we can only crudely discern from electrophysiological cues and the brain morphology in which circuits the activity arises. For example, we cannot assess contributions of small or large field neurons [11,15-20] that should participate in object tracking or flow processing, respectively, on these results. Nonetheless, the miniature electrodes are more robust and less damaging to the circuitry than alternative approaches, such as calcium-imaging or patch-clamping that require tissue-free recording paths. But most importantly, these electrodes can provide long-lasting stable recordings. Our results make it clear that their spatio-temporal properties are sufficient to characterize general changes in activity of the optic lobes during the stimulus selection behavior.

In the *Drosophila* brain, we could not find other sites - outside the possible lobula plates - that responded reliably with action potentials (spikes) to visual motion. It was also difficult to find other sites that generated reasonably large spikes; the sites were typically silent, or they fired sparsely with small spikes, uncorrelated to motion stimuli. When the electrode rested favorably within the optic lobe, we carefully tested that the reference electrode (of low impedance) did not influence the recorded activity. Once suitable spikes to a test stimulus (motion field) were picked up, we moved the location and depth of the reference electrode. Because this had no effect on the responses and the output of the two recording electrodes differed from each other, we knew that the spikes came were intended. If instead the spikes had been picked up by the reference electrode, the recording electrodes should have showed similar patterning. This we never saw.

These observations also made it clear that was quite improbable that the electrodes moved within the brain during the torque responses, or that some hypothetical movements of the electrodes were the source for the modulation. Even if a fly’s head was somehow able to move in the torque meter (which it did not in all practical purposes; see **Video S1**), these ultra-light and free-standing electrodes would move with it, not against it (**Fig. 5A**). Thus, for any motion artifacts to modulate the output of two electrodes, so as to match their separate patterns of activity, the left and right brain hemispheres would have needed to be moving in relative independence inside the head capsule, yet in still synchrony with a fly’s torque responses. The difficulty of these conjectures, and the finding that the mean firing rate of the neurons often remained stable throughout minutes-long recordings, makes it unlikely that the changes in the neural activity were caused by moving electrodes. While fast rhythmic muscle contractions (“clock-spikes”) within the head capsule may participate in refining the light input [21-23] when a fly orients between the opposing stimuli, particularly in 5-6 Hz range as reported for *Musca* in a near free-flight conditions [24], such actions must happen under neural control. Here, they would need to be driven in conjunction with *Drosophila*’s choices that have a 20-100-times slower periodicity (**Figs. 6A** and **2A**; more below).

Therefore, the observed modulation in the neural activity was almost certainly generated intrinsically, either within the optic lobes or within the brain proper that links the two eyes.

**III. CONSIDERING DIFFERENT VIEWS**

**III.1. unstable flight motor equilibrium**

Automatic steering reflexes help a fly to control its flight path in changing environment [10]. Some of these reflexes are protective, and thus may have the strength to override the behavior a fly is engaged in. In particular, it has been shown by different experimental assays that large-field expansion elicits high magnitude and *tightly phase-locked* optomotor responses away from the pole of expansion. Hence we ask: could the square-waved “torque-turns” and the intrinsic modulation, which we report here as signs of selective orientation, be outcomes of unstable steering reflexes, evoked by competitive stimuli? We next consider evidence for steering-reflex hypothesis, before presenting other evidence, which seems more difficult to explain by this tenet in our experimental conditions.

***III.1.1. Steering-reflex hypothesis: Behavior***

In response to an expanding flow field, *Drosophila* show extremely robust optomotor steering reflexes [4,11,12,25,26]. An expanding flow field generates very strong optomotor responses. Centering the focus of expansion frontally, generates a highly unstable equilibrium - if the frontal pole moves slightly to one side, the steering response is immediately and powerfully in the other direction. Thus, when the fly is given control to steer the pole, it *always* turns to face the contracting pole.

We first tested how flies responded to frontally expanding flow fields by using a fiber optic display (*i.e.* two panoramic hemifields of vertical stripes moving left and right). We found that frontal field expansion from a single central pole evoked strong left or right orienting responses. But for thick stripes (λ = 40^o^) these responses were intermixed with separate landing responses toward the pole of expansion (the flies extended their legs up front), whereas narrower stripes (λ = 30^o^) evoked only turns. Thus, the behavior seemed to depend upon how close the fly “perceived” the centre of expansion to be. These findings differ from those of Tammero and Dickinson [25], who used an expanding object as a stimulus. They found that when an object expanded within the frontal field of view, *Drosophila* typically elicited leg extensions without a steering response. Nonetheless, these results could be interpreted as signs of unstable steering-reflex to avoid collision.

We next blocked both the frontal pole of expansion and the rear pole of contraction with black (or white) cardboard cuttings of various sizes, thereby optically isolating the left and right moving scenes. The results from these experiments present a bigger challenge to a simple steering-reflex explanation.

***III.1.1.1. Behavioral Evidence Challenging the Steering-Reflex Hypothesis***.

1. Because both the frontal and ventral components of the visual motion scenes were missing (Materials and Methods), it was clear that our flight simulator did not generate an obvious frontal field expansion; thus, the spatially isolated left and right moving scenes were very different from the spatially continuous visual input used to test frontal expansion in [4,11,12,25,26]. Hence the resulting torque responses could not have been evoked by expansion in the frontal pole because there really was no clear ‘frontal pole’ for the flies to see. Furthermore, regardless of the stimulus patterns used in this assay, bilateral stimuli never evoked landing responses; unlike the case when the frontally expanding pole was visible to the fly (see above).
2. We found that a fly could exert is torque responses between the left and right scenes even during back-to-front motion. These responses were much weaker, mostly containing saccade-like events (**Fig. S4**). Importantly, however, even though when a fly faced the contracting pole of the flow field, this did not quench its selective (exploratory) visual behavior.
3. Smaller bilateral stimuli did not alter the amplitudes of the torque responses nor their dynamics considerably (**Fig. S5**). This finding is consistent with the earlier study that used small objects (vertical bars) to investigate changes in yaw torque in relation to competing visual motion stimuli [1].
4. Fruit flies sometimes mixed left and right torque responses with periods of flying straight (**Figs S3, S5** and **S6**). This finding implies that - although the switch-like dynamics of torque responses seem suggestively reflect-like -, the flies can withhold from responding to the competitive stimuli at any one moment.
5. We found that unsymmetrical input (i.e. moving the fly in respect to the left and right scenes or dephasing the movements of the scenes) did not change the dynamics of the torque responses noticeably. This finding opposes directly the steering-reflex hypothesis in our stimulus paradigm, in which case the torque responses should have been strictly toward the side further away from the centre of the field expansion (*i.e.* the expanding pole).
6. The finding that there was a long latency before a fly chooses the side from which to modulate its yaw torque, and the variability of these choices (**Fig. 3**), is against the torque responses resulting from the flies simply trying to avoid a head-on collision. We estimated that the fly’s central brain obtains visual motion information within 30 ms from the stimulus onset (**Fig. 5**). If a fly waited further several hundreds of milliseconds, as happens here, before exerting a protective torque response, it may find its end with the rapidly approaching object/predator.
7. The flies could also respond against to unilateral motion scenes (**Fig. S7**). This observation suggests that in our stimulus paradigm the flies indeed choose which motion scene to pursue.
8. Visual motion input is the same for different flies, yet they responded with individually patterned torque responses (duration and intervals). Thus, the responses were far from simple reflex-like - all or nothing events -, but had highly variable time-courses and patterning (*cf*. **Figs. 2** and **S2D**).
9. From the results shown in **Figs. 3** and **5,** we suggest that the prolonged delay is a typical attribute of the competing stimuli paradigm. However, the same delay can obviously also happen even without any competing motion stimulus, as seen from the red torque trace in **Fig. S7A** (delay ~110 ms), and from the blue trace of **Fig. S7B**, where the delay between onset of the (unilateral) motion amounts to about 700 ms. Such variability suggests that even during unilateral stimulation, the flies decide when they exert their torque response; this degree of spontaneity is very different from what we see in a classical reflex (**Fig. 2D**).

***Summary*:** Based on these findings and observations, it appears that the torque responses in the competing stimuli paradigm may resist simple generalizing interpretations for their initiation. Here, our consensus estimate is that during this paradigm, different aspects of neural processing for both object tracking and avoidance is perhaps blended to generate these results.

Nonetheless, it is important to realize that whether the torque responses resulted from stimulus attraction, repulsion, or other cause, this has relatively little to do with the two main messages of this article, which are:

(1) By using the simple competing stimulus paradigm, we could specify torque responses as binary left or right choices, and correlate them to neural activity in the left and right optic lobes.

(2) The orienting behavior and neural responses together made a highly suggestive case that intrinsic activity in the *Drosophila* brain modulates visual information processing when it decides to turn.

**V. NEURAL ACTIVITY**

**IV.1. Differences between Firing patterns and LFPs**

We pool data from experiments, which contained switch-like torque responses of roughly equal size and duration to left and right, to compare how *Drosophila*’s choice of left or right visual stimulus affects the neural activity in the optic lobes. Again, we remind the reader that because the tethered flies are firmly held to the torque-meter [8], the visual input to their eyes during the torque responses remain the same (**Materials and Methods**).

***Local information processing***: By subtracting the neural activity during left and right torque responses (or stimulus choices) in one side (measured by a single electrode; left or right), we find that the neural firing patterns of local neurons come in two major classes (**Fig. S8A**). They show either ipsi- or contralateral ocular dominance to visual motion, in similar numbers (*i.e.* neurons that fire more during ipsi- [blue] or contralateral [red] torque responses). By their temporal activity patters, these neurons can be further sorted into two groups: transiently firing and more slowly firing neurons of ipsi- or contralateral preference.

***Global information processing***: On the other hand, similar comparison for the left and right LFPs during left or right torque responses shows that the global neural activity in the left side of the brain always increases during the left choices and the neural activity in the right optic lobe always increases during the right choices. This ipsilateral global preference of LFPs was true for all the analyzed experiments (**Fig. S8B**), suggesting that the global activity of the optic lobes is gated in respect to the fly’s orientation choices.

**IV.2. Changes in power spectra of LFPs during opposite torque responses**

We estimated the relative changes in the frequency content of LFPs in this way:

1. LFP recordings from the right (blue) or left (red) optic lobes were aligned, cut and pooled for each switch-like torque response, using the zero-crossings in the yaw torque as start-points (**Fig. S9A**, the left edge of the gray and light gray bars).
2. These LFP traces were then trimmed to a suitable size, typically lasting about 60% of the average torque response (**Fig. S9A**, black; area within the gray bars). With 1 kHz sampling, the traces had usually 3,000-5,000 points.
3. As this experiment consisted of 14 torque responses to left and 14 responses to right (**Fig. S9A**), each optic lobe (or electrode) is represented with two matrixes: one containing traces for the left choices and the other for the right choices; thus we have four [14 x 4,096] matrixes. The means of such matrixes, or average signals, are shown in blue, navy, red and wine (**A**).
4. We calculate the power spectra for each 4,096 points-long LFP trace (n = 14), using 1024-point samples with 50% overlap. For each trace, this gives 7 spectral samples that are averaged. These average samples are then averaged across each matrix giving the mean power spectral estimate of LFP during one-sided torque response in one optic lobe (n = 7 x 14 = 98; **Fig. S9B**).
5. Thus, we obtain two mean power spectral estimates of LFPs for each optic lobe (or electrode). One for the right choice and the other for the left choice. To see if the neural activity in the right side of the brain was increased during the right torque responses, we subtract the spectra E#1_R_-E#1_L_. For the differences in the neural activity in the left side of the brain, we subtract the spectra E#2_L_-E#2_R_. These differences are then given as relative changes: 100*(E#1_R_-E#1_L_)/-E#1_L_ and 100*(E#1_L_-E#1_R_)/-E#1_R_ (**Fig. S8C**).

LFP recordings (**Figs. 8**, **9**, **S9** and **S10**) show that outputs of the left and right optic lobes are dynamically modulated with a fly’s choices between the left and right moving scenes. To characterize this modulation further, we calculated the power-indexes for the LFPs in the left and right optic lobes during the visual choice paradigm. The rationale and details of such calculations are shown in **Fig. S10**. The results from single experiments (such as **D*-*E** and **F*-*G**) and those pooled from 5 different flies (**Figs. 9A-B**) show that the outputs of the optic lobes seem to be gated simultaneously by separate inhibitory and excitatory processes. The output of the left optic lobe is enhanced during left choices (torque responses) and suppressed during right choices, whereas the output of the right optic lobe is enhanced during right choices and suppressed during left choices. The dynamics for the ipsilateral enhancement are faster than for the contralateral suppression.

**V. Could intrinsic modulation arise from the thoracic ganglia?**

The haltere sensory fields send anterior projections into the central brain, converging upon pre-motor centers housing lobula plate tangential cell axons [27,28]. There is ample electrophysiological evidence that the haltere sensory system directly activates steering motoneurons of the wings [10], and receives direct visual input [29]. Thus, a reflex arc between visual input, the halteres, and the wings might explain how a fly commands a turn [13]. Since the haltere input also projects to the brain [27,30], it may modify network activity there. Perhaps this could manifest as changes in the field potentials?

Whilst these factors might be important in flight control, their feed upon LFP dynamics is probably small for two reasons. First, the visual motion input and the neural responses of the optic lobes precede the switch-like torque responses (**Figs. 5, 9, S10**). As the visual motion input for the eyes remains the same throughout the experiment, it seems unlikely that within these stationary conditions (for both visual and mechanosensory inputs) the haltere output would start to modify the neural activity of the optic lobes before the fly actually executes its major steering response. Second, when a fly rests, its halteres should not modulate neural activity of the brain in the same way as they do when the fly is flying. However, the intrinsic modulation in the neural activity of the optic lobes is quite similar in resting and flying flies; for one-sided visual motion (**Figs. 7C-D**), and when exerting torque responses toward the same-sided visual motion (**Figs. 7A-B**), respectively.

**VI. REFERENCES**

1. Wolf R, Heisenberg M (1980) On the fine structure of yaw torque in visual flight orientation of *Drosophila melanogaster* J Comp Physiol 140: 69-80.

2. Tang S, Wolf R, Xu S, Heisenberg M (2004) Visual pattern recognition in *Drosophila* is invariant for retinal position. Science 305: 1020-1022.

3. Tang S, Guo A (2001) Choice behavior of *Drosophila* facing contradictory visual cues. Science 294: 1543-1547.

4. Tammero LF, Dickinson MH (2002) The influence of visual landscape on the free flight behavior of the fruit fly *Drosophila melanogaster*. J Exp Biol 205: 327-343.

5. Heisenberg M, Wolf R (1979) On the Fine Structure of Yaw Torque in Visual Flight Orientation of *Drosophila melanogaster*. J Comp Physiol 130: 113-130.

6. Heisenberg M, Wolf R (1984) Selective attention. Vision in *Drosophila* Genetics of microbehaviour: Springer-Verlag. pp. 183-193.

7. Heisenberg M, Wolf R (1993) The sensory-motor link in motion-dependent flight-control of flies. In: Miles FA, Wallman J, editors. Visual motion and its role in the stabilization of gaze. Amsterdam: Elsevier. pp. 265-283.

8. Götz KG (1964) 0ptomotorische Untersuchung des visuellen Systems einiger Augenmutanten der Fruehtfliege *Drosophila*. Kybernetik 2: 77-92.

9. Götz KG (1975) The optomotor equilibrium of the *Drosophila* navigation system. J Comp Physiol 99: 187-210.

10. Frye MA, Dickinson MH (2004) Closing the loop between neurobiology and flight behavior in *Drosophila*. Curr Opin Neurobiol 14: 729-736.

11. Duistermars BJ, Reiser MB, Zhu Y, Frye MA (2007) Dynamic properties of large-field and small-field optomotor flight responses in *Drosophila*. J Comp Physiol A 193: 787-799.

12. Duistermars BJ, Chow DM, Condro M, Frye MA (2007) The spatial, temporal and contrast properties of expansion and rotation flight optomotor responses in *Drosophila*. J Exp Biol 210: 3218-3227.

13. Dickinson M (2006) Insect flight. Curr Biol 16: R309-314.

14. Knudsen EI (2007) Fundamental components of attention. Annu Rev Neurosci 30: 57-78.

15. Egelhaaf M, Hausen K, Reichardt W, Wehrhahn C (1988) Visual course control in flies relies on neuronal computation of object and background motion. Trends Neurosci 11: 351-358.

16. Egelhaaf M (1985) On the neuronal basis of figure-ground discrimination by relative motion in the visual-system of the fly .1. Behavioral constraints imposed on the neuronal network and the role of the optomotor system. Biol Cybern 52: 123-140.

17. Egelhaaf M (1985) On the neuronal basis of figure-ground discrimination by relative motion in the visual-system of the fly .2. Figure-detection cells, a new class of visual interneurones. Biol Cybern 52: 195-209.

18. Egelhaaf M (1985) On the neuronal basis of figure-ground discrimination by relative motion in the visual-system of the fly .3. Possible input circuitries and behavioral significance of the fd-cells. Biol Cybern 52: 267-280

19. Kimmerle B, Egelhaaf M (2000) Performance of fly visual interneurons during object fixation. J Neurosci 20: 6256-6266.

20. Bolzon DM, Nordstrom K, O'Carroll DC (2009) Local and large-range inhibition in feature detection. J Neurosci 29: 14143-14150.

21. Hengstenberg R (1971) Das Augenmuskelsystem der Stubenfliege *Musca domestica*. Biological Cybernetics 9: 56-77.

22. Patterson J (1973) The eye muscle of *Calliphora vomitoria*. l. Spontaneous activity and the effects of light and dark adaptation. J Exp Biol 58: 565-583.

23. Lowne BT (1890) The Anatomy, Physiology, Morphology and Development of the Blowfly (*Calliphora erythrocephala*); Porter RH, editor. London

24. Franceschini N, Chagneux R (1997) Repetitive scanning in the fly compound eye. In: Eisner N, Wassle H, editors. Proceedings of the 25th Gottingen Neurobiology Conference 1997; Volume II. Stuttgart, New York: Georg Thieme Verlag. pp. 279.

25. Tammero LF, Dickinson MH (2002) Collision-avoidance and landing responses are mediated by separate pathways in the fruit fly, *Drosophila melanogaster*. J Exp Biol 205: 2785-2798.

26. Tammero LF, Frye MA, Dickinson MH (2004) Spatial organization of visuomotor reflexes in *Drosophila*. J Exp Biol 207: 113-122.

27. Strausfeld NJ, Seyan HS (1985) Convergence of visual, haltere, and prosternal inputs at neck motor neurons of *Calliphora eryhrocephala*. Cell Tissue Res 240: 601-615.

28. Sandeman DC, Markl H (1980) Head movement in flies (*Calliphora*) produced by deflexion of the halteres. J Exp Biol 85: 43-60.

29. Chan WP, Prete F, Dickinson MH (1998) Visual input to the efferent control system of a fly's "gyroscope". Science 280: 289-292.

30. Chan WP, Dickinson MH (1996) Position-specific central projections of mechanosensory neurons on the haltere of the blow fly, *Calliphora vicina*. J Comp Neurol 369: 405-418.
