# Supplementary figures and images for: Intrinsic Activity in the Fly Brain Gates Visual Information during Behavioral Choices
Source: PLoS One. 2010 Dec 30;5(12):e14455. doi: 10.1371/journal.pone.0014455 (PMC3012687; doi:10.1371/journal.pone.0014455)

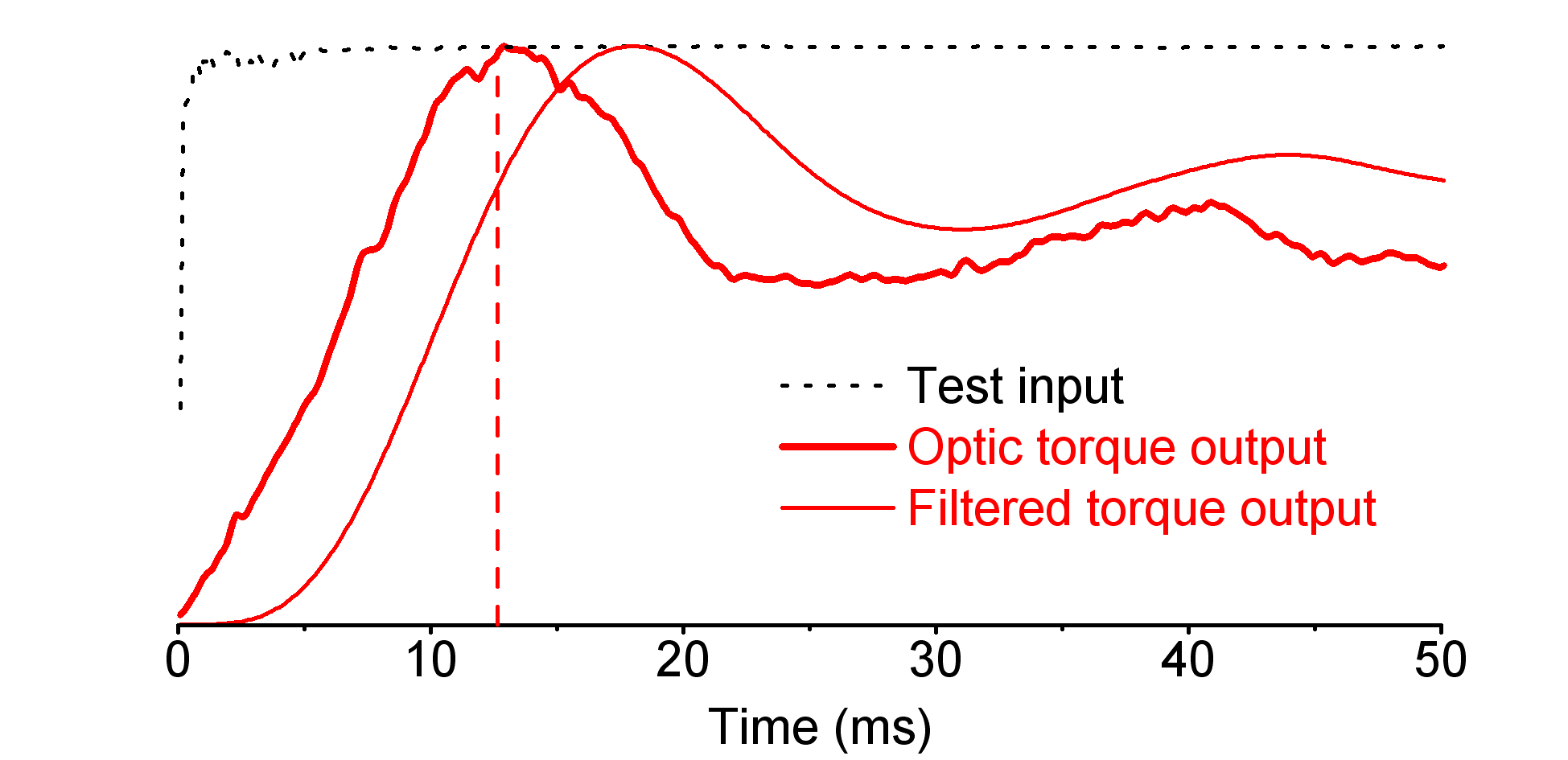

Supplement: Figure S1 — Output of the torque meter to an electro-magnetic pulse (input) shows a fast rise-time and signal-to-noise ratio. The rise time is 12.5 ms (red dotted line). (0.08 MB TIF) [file pone.0014455.s002.tif]

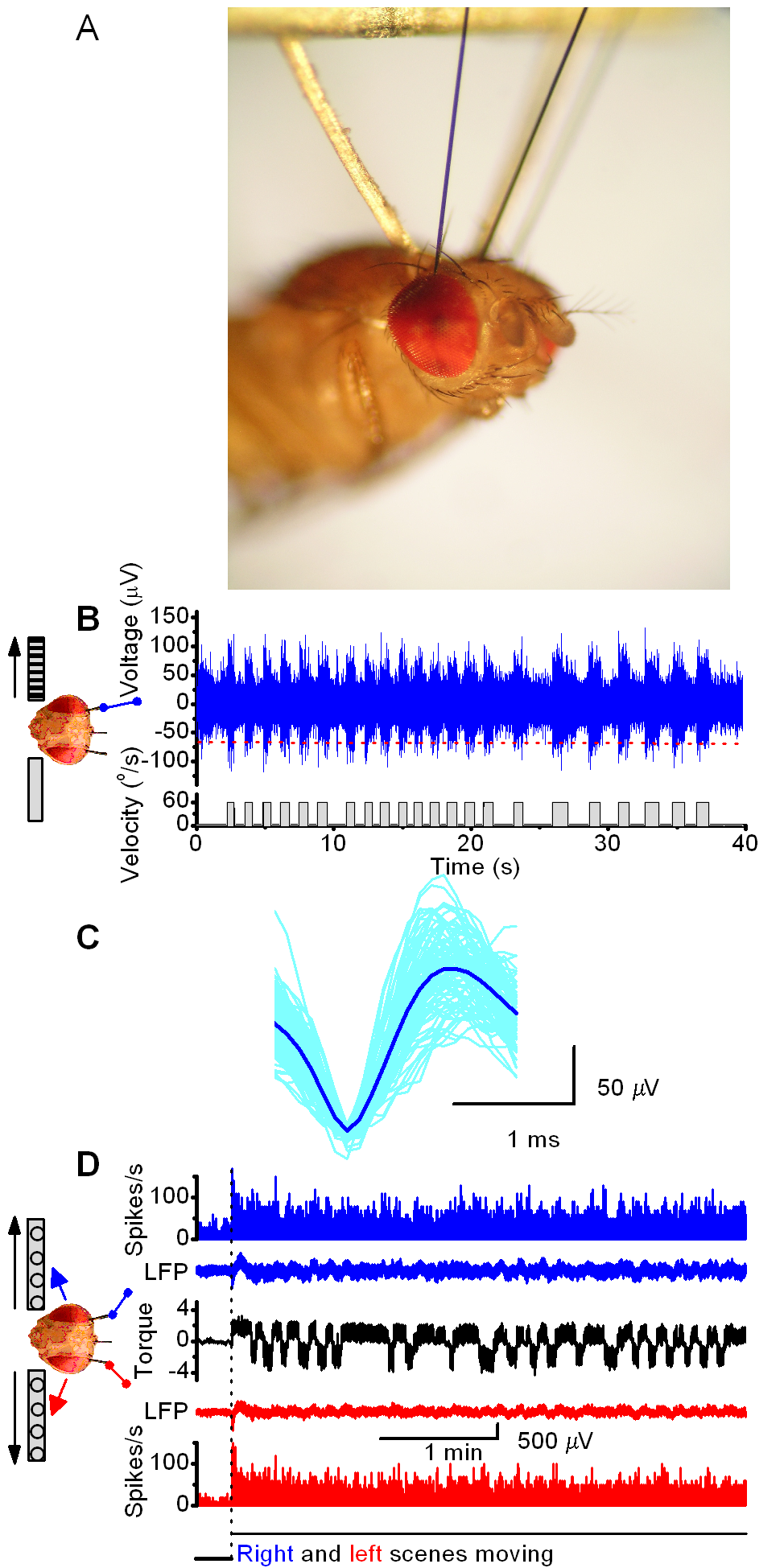

Supplement: Figure S2 — Spike detection, and orientation with different competing stimuli. (A) The standardized positions for the recording electrodes in the head of a flying Drosophila. The small harness, glued between the head and body (the thicker silver wires), was used to clamp the fly's head in a fixed position and orientation. The miniaturized electrodes are the black thin wires. Electrical responses from a fly's optic lobe (most likely from the lobula plate) to a single moving field, recorded with a miniaturized tungsten electrode. (B) Continuous voltage signals (blue) during a constant velocity motion (stripes) stimulus on the right scene (light grey, below) recorded from the right optic lobe of a resting (non-flying) fly. The spikes are detected by a threshold (red dotted line). (C) Mean spike (blue) and individual spikes (light cyan). (D) Characteristic behavior (yaw torque, black) and neural responses in the optic lobes (firing rates, above; LFPs, below) to o-patterns that move to opposing directions at 60°/s; recorded form tethered flying Drosophila. During the experiments both eyes receive continuous motion stimuli, as indicated by the fly-head cartoon. Similar to stripe-patterns (cf. Fig. 6), the fly attempts to follow either a left or right moving scene at any one time (i.e. generating torque responses to left or right); the neural output of its optic lobes (right, blue; left, red) are tuned with these responses. Notice the variable time courses of the left and right torque responses of this fly; compare the behavior of five other flies in Fig. 6 and S3. (5.70 MB TIF) [file pone.0014455.s003.tif]

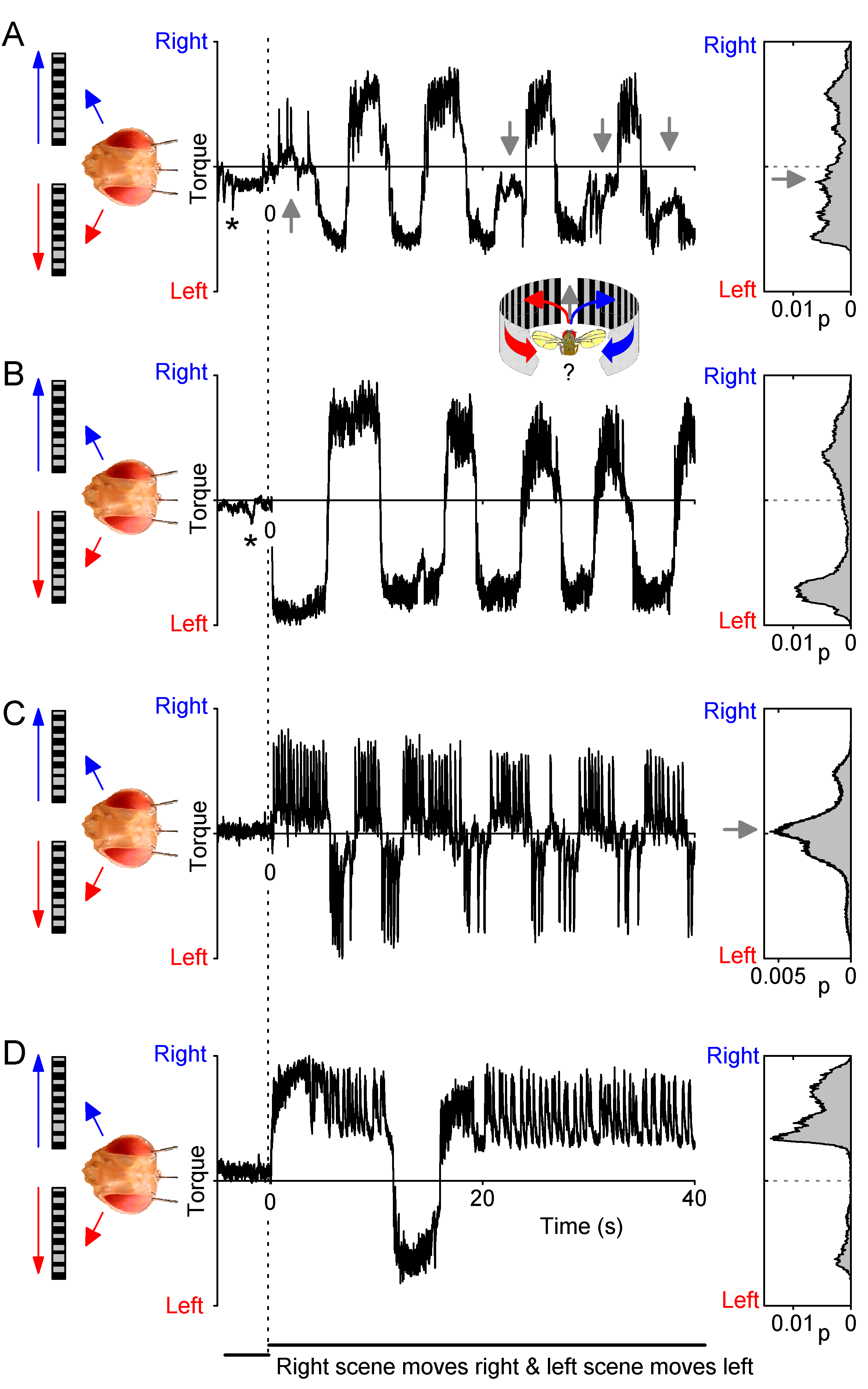

Supplement: Figure S3 — In competing stimuli paradigm, the dynamics of the switch-like visual selection vary from fly to fly. The figure shows the orienting behavior of four different flies during bilateral visual motion at 60°/s. Before the motion stimuli, the tethered flying flies can generate exploratory saccades to left and right (stars). When the scenes move, the flies begin to generate larger side-to-side flipping torque responses toward the left (down) or right stimulus (up), one at a time. Intermixed with these responses, some flies also orient straight for brief periods (gray arrows). (A) A fly with a 3-state orientation responses; these mostly flipped between the left and right scenes, but it also flew straight frequently. Interestingly, from the stimulus onset, this Drosophila flew straight for about 4 seconds (apart from few saccades to right) before first choosing the left stimulus. (B) A fly that generated rather symmetrical torque responses between the left and right scenes with a slight preference to the left scene. (C) A fly whose torque responses transiently flipped between the left and the right scenes, but it oriented mostly straight. (D) A fly that generated symmetrical torque responses between the scenes, but chose to orient mostly toward the right scene. The insets show the corresponding probability density functions. (1.34 MB TIF) [file pone.0014455.s004.tif]

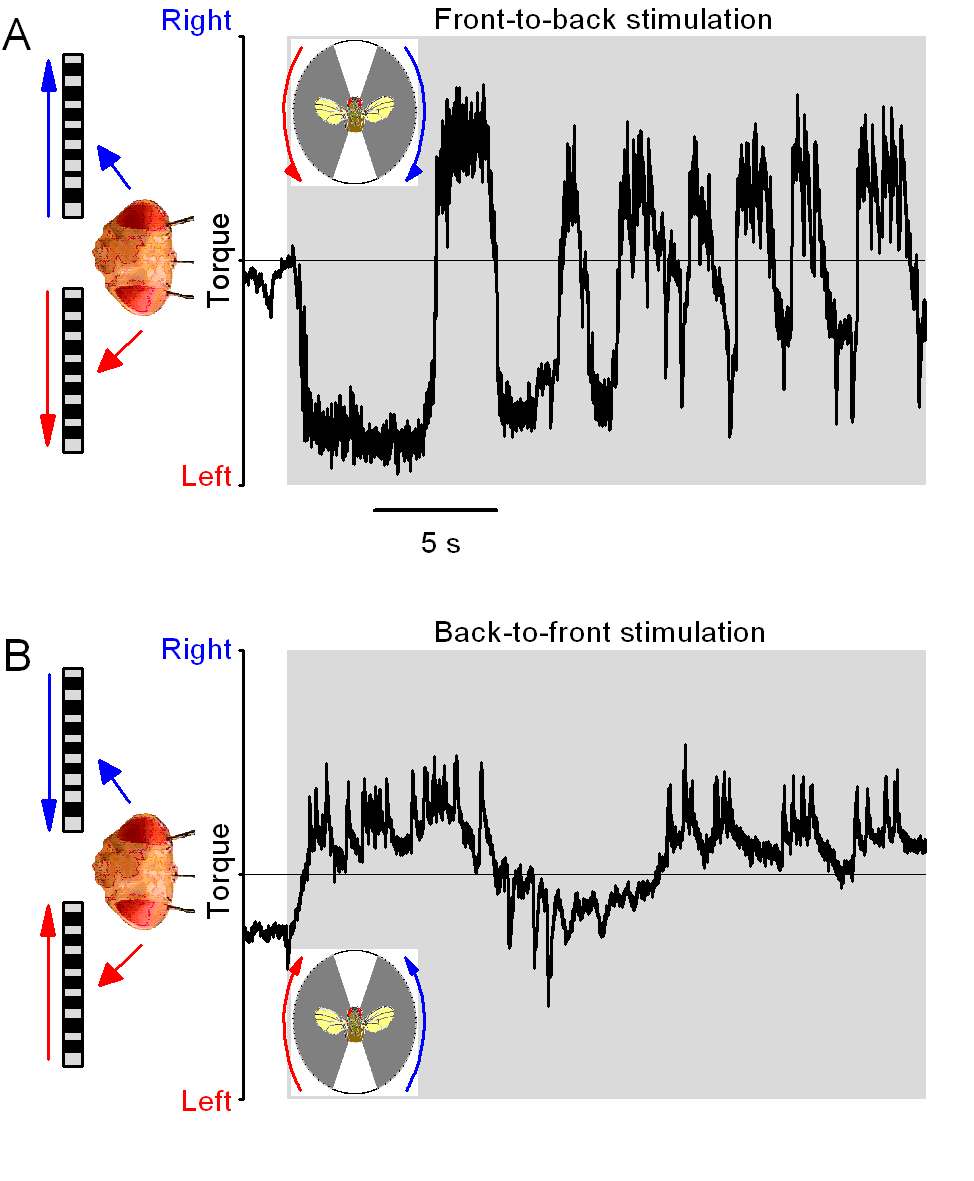

Supplement: Figure S4 — In competing stimuli paradigm, a fly's switch-like orienting between the left or right stimuli is evoked efficiently by front-to-back motion. The figure shows traces of behavior (yaw torque) of the same tethered flying Drosophila to (A) front-to-back and (B) back-to-front motion (bilateral stripe scenes: azimuth ±150°, elevation ±40°, velocity 60°/s). When the scenes are moving (light gray areas), the fly begins to exert torque responses between the left (down) and right scenes (up). These responses are much stronger for the front-to-back than for the back-to-front motion. (3.46 MB TIF) [file pone.0014455.s005.tif]

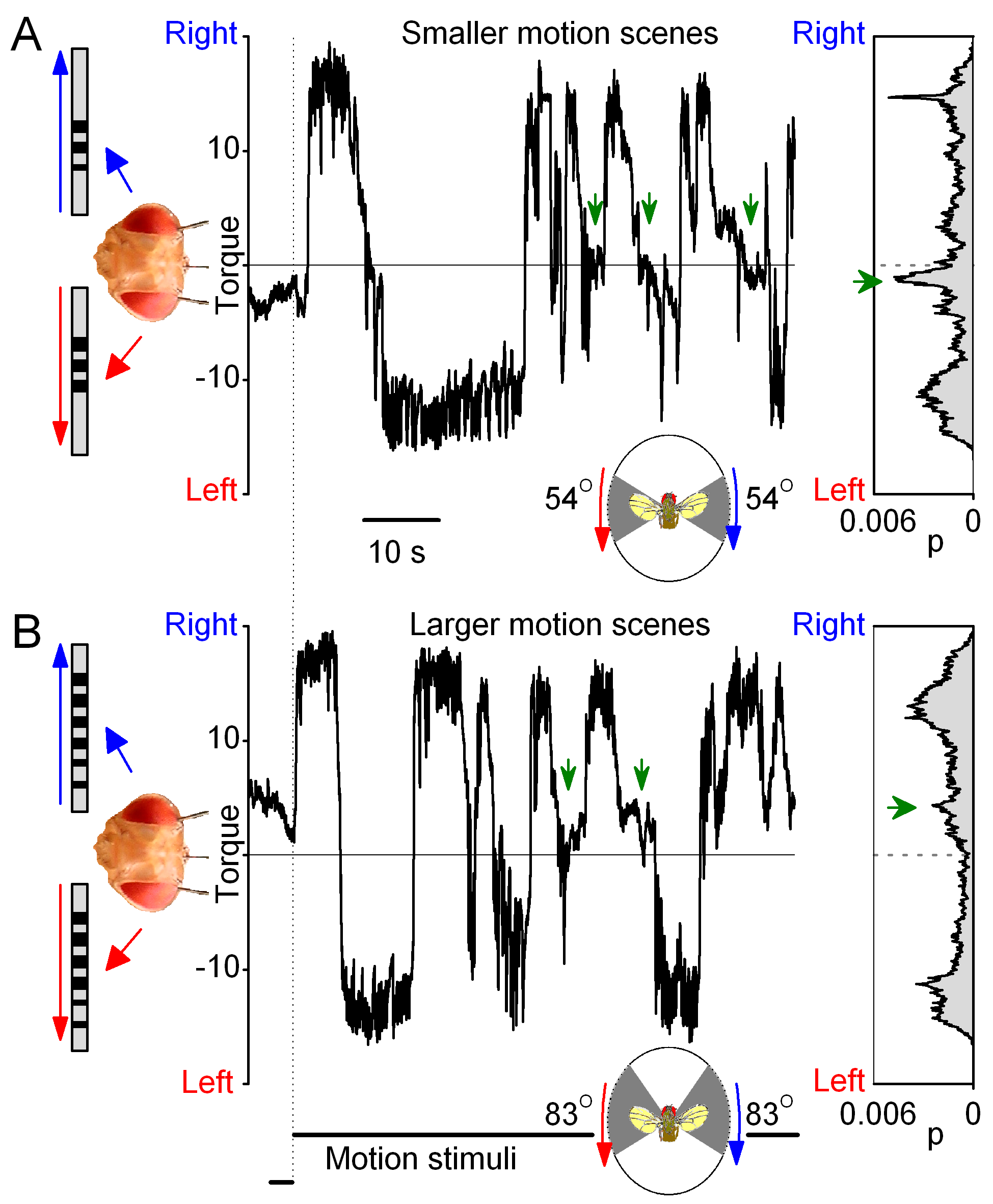

Supplement: Figure S5 — In competing stimuli paradigm, a fly's switch-like orienting occurs similarly for different sizes of motion scenes (cf. Fig. S6). The figure shows the behavior of the same tethered flying Drosophila to smaller and bigger stimuli (gray sectors in the circular inserts): (A) at 54° lateral scenes (126° in the front and back blacked out), and (B) 83° (69° blacked out frontally and 125° dorsally), respectively. In both cases, when the scenes are set in motion (60°/s; dotted line), there is a variable delay before the fly starts to generate side-to-side flipping torque responses, attempting to rotate toward the left (down) or right stimulus (up). Intermixed with these responses are periods of flying straight (approximately zero torque; green arrow heads), implying that the fly can also oppose the stimuli. Here, the field size of stimulation seems to have little influence on the size of torque responses. These results imply that torque responses are not evoked by some specific features in the stimulus patterns, but that they define the fly's choices. Insets show the corresponding probability density functions. (0.73 MB TIF) [file pone.0014455.s006.tif]

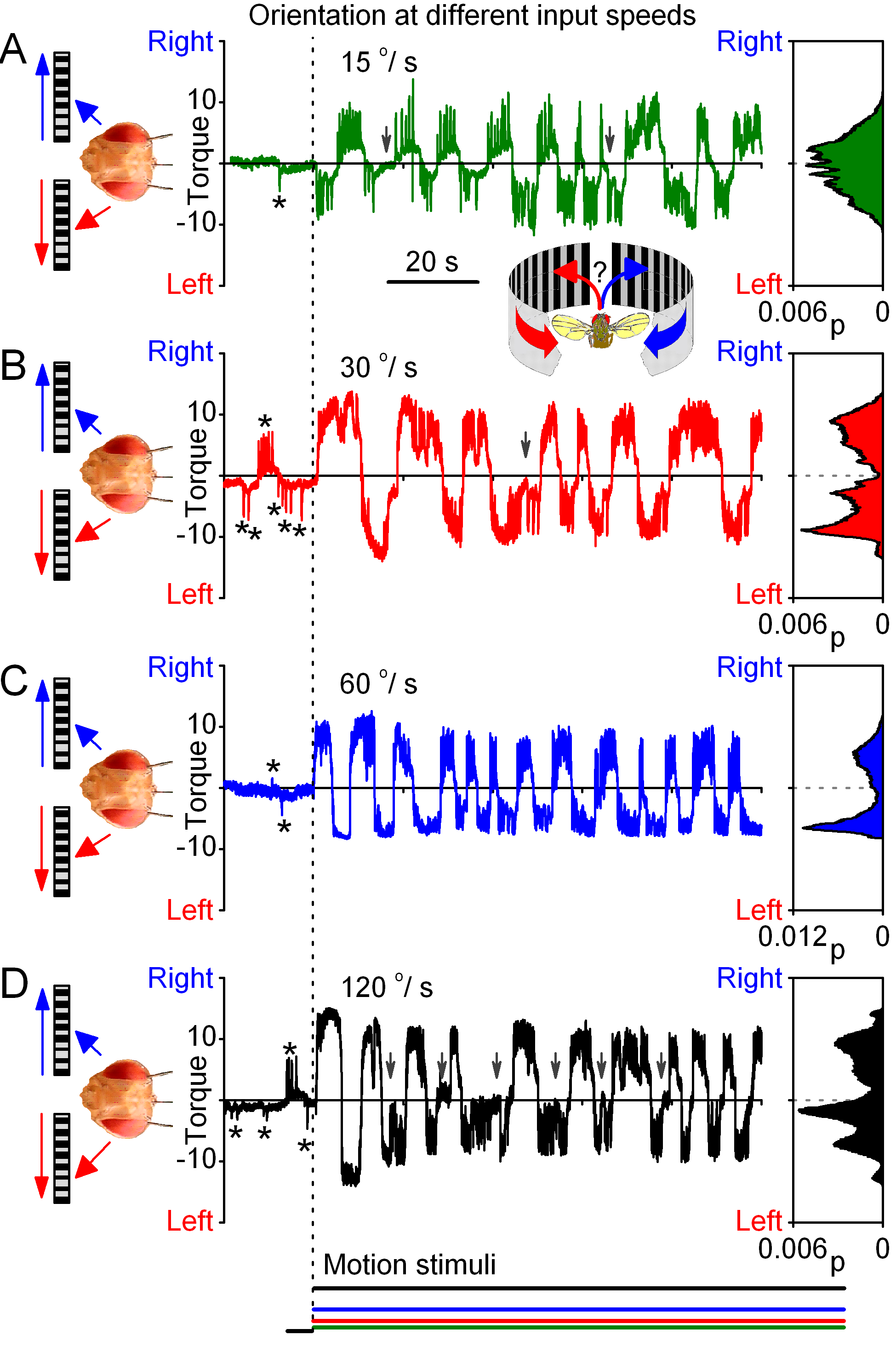

Supplement: Figure S6 — Visual selection occurs switch-like at different speeds of competing stimuli (150° left and right). The figure shows the orienting of the same tethered flying Drosophila during competing visual stimuli paradigm at different speeds: (A) at 15°/s, (B) 30°/s, (C) 60°/s, and (D) at 120°/s. Before the motion stimuli, the fly makes characteristic exploratory saccades to left and right (stars). When the scenes are set in motion, the fly generates larger side-to-side flipping torque responses to the left (down) or right stimulus (up), but it also can orient straight (small gray arrows). Although increasing the speed of stimulation appears to amplify the torque responses (cf. A and D), this evokes no obvious/trivial causal changes on their duration and frequency. These results imply that the switch-like orienting is not heavily dependent on the stimulus speed. Insets show the corresponding probability density functions. (1.93 MB TIF) [file pone.0014455.s007.tif]

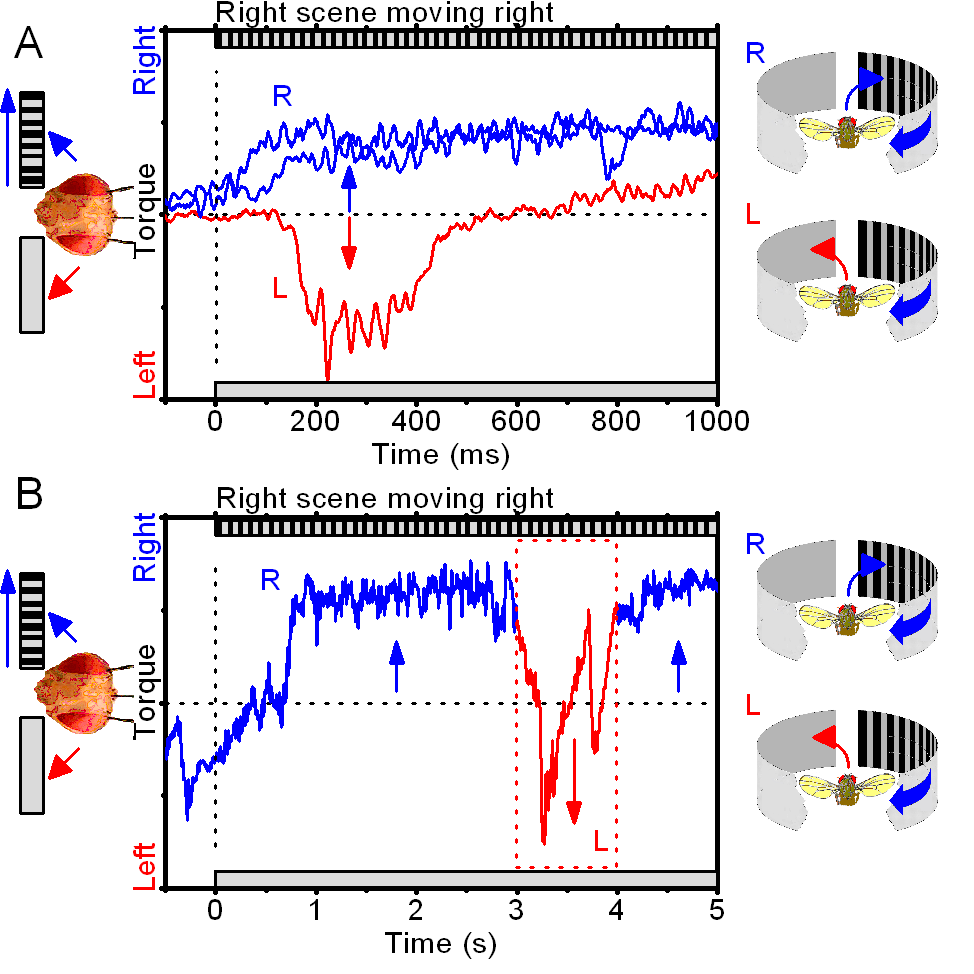

Supplement: Figure S7 — A tethered flying Drosophila can react against unilateral motion field. Although the right scene, R, moves, the fly exert its torque response to the opposite, unmoving side (the blank left scene, L). (A) Even at the onset of the unilateral motion, a fly may occasionally respond against it by generating a torque response toward the opposing blank scene. (B) Also when engaged in pursuing the unilateral motion, a fly sometimes briefly interrupts its response to orient toward the unmoving blank scene. This unexpected behavior highlights that even if its reactions are triggered by a strong moving field-stimulus, these can be modified at any one time by internal motor commands. (2.85 MB TIF) [file pone.0014455.s008.tif]

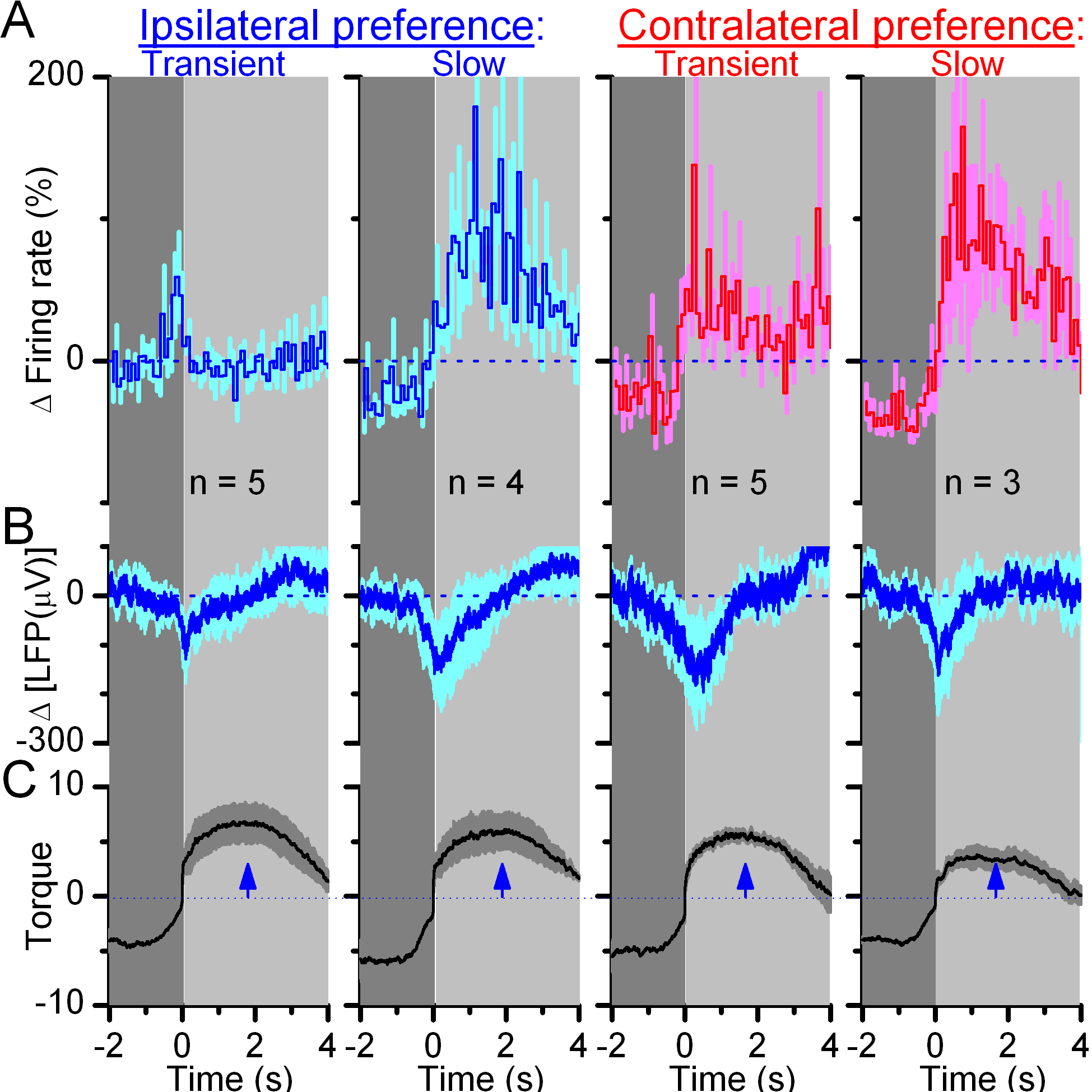

Supplement: Figure S8 — Differences in local and global neural activity. Firing patterns of individual neurons (A) in the optic lobes show variable tuning for ipsi- (blue traces) and contralateral (red traces) visual motion, (B) but the global activity of the optic lobes (LFPs) always increases when selecting (torque responses toward) ipsilateral scenes. Except for torque (C), traces show the relative change in responses toward the preferred side; aligned by the torque responses. By their preference for ipsi- or contralateral stimuli, the neurons in the LPs can be sorted into four groups (data from 9 flies, from 17 neurons [thus from 17 different electrodes]; bin-size 100 ms). Transient increase neurons fire the most at the early part of the fly's torque response to ipsilateral objects. Slow increase neurons steadily increase their firing rate, until the ipsilateral torque response starts to ease off. Transient decrease neurons respond best at the beginning of a shift to contralateral side. Slow decrease neurons are most active when the torque response toward the contralateral stimulus peak. Unlike the firing rates, the absolute amplitudes of the corresponding LFPs always reach their maxima (i.e. points downwards) during ipsilateral viewing. Means ± SEMs shown. (0.30 MB TIF) [file pone.0014455.s009.tif]

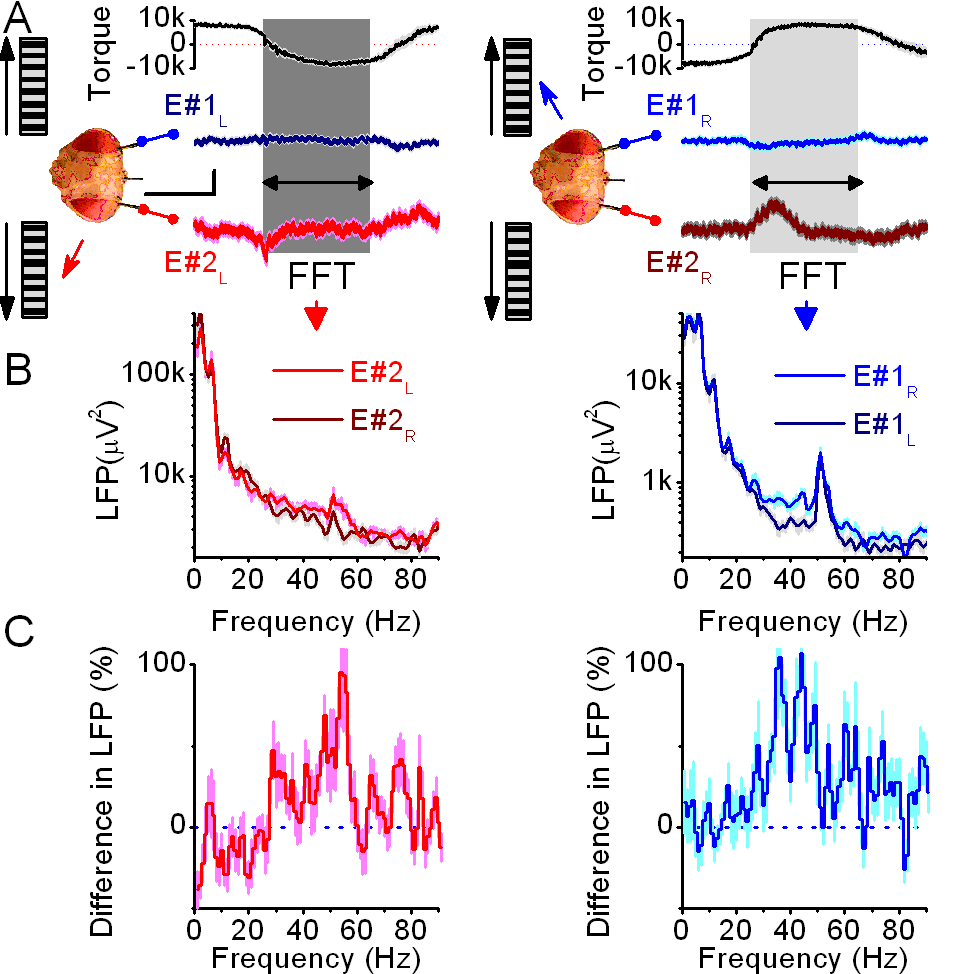

Supplement: Figure S9 — Ipsilateral neural activity is enhanced during visual choice paradigm. This figure shows typical changes in the power spectra of LFPs, as measured from the optic lobes during left and right torque responses of a single fly. (A) The fly generated 14 switch-like torque responses to left and right during bilateral motion stimulus (stripe-bars). Mean torque responses (black) to left (down) and right (up) are aligned with the corresponding mean LFPs, measured by the right (E#1, blue) and left electrode (E#2, red). Scale: 2 s/300 µV. Gray areas indicate the sections across LFP recordings that were used for calculating the power spectral estimates. (B) Power spectral estimates (mean ± SEM, n = 98 samples) for the left optic lobe (left) and the right optic lobe (right) during left (red and navy LFPs) and right (blue and wine LFPs) choices. (C) Relative differences in the neural activity in the left (red) and right (blue) optic lobes during the left and right choices (mean ± SEM, n = 14 torque responses). Similar to the pooled data across the flies (Fig. 8B), here the ipsilateral signals are boosted at ∼20–90 Hz. (2.85 MB TIF) [file pone.0014455.s010.tif]

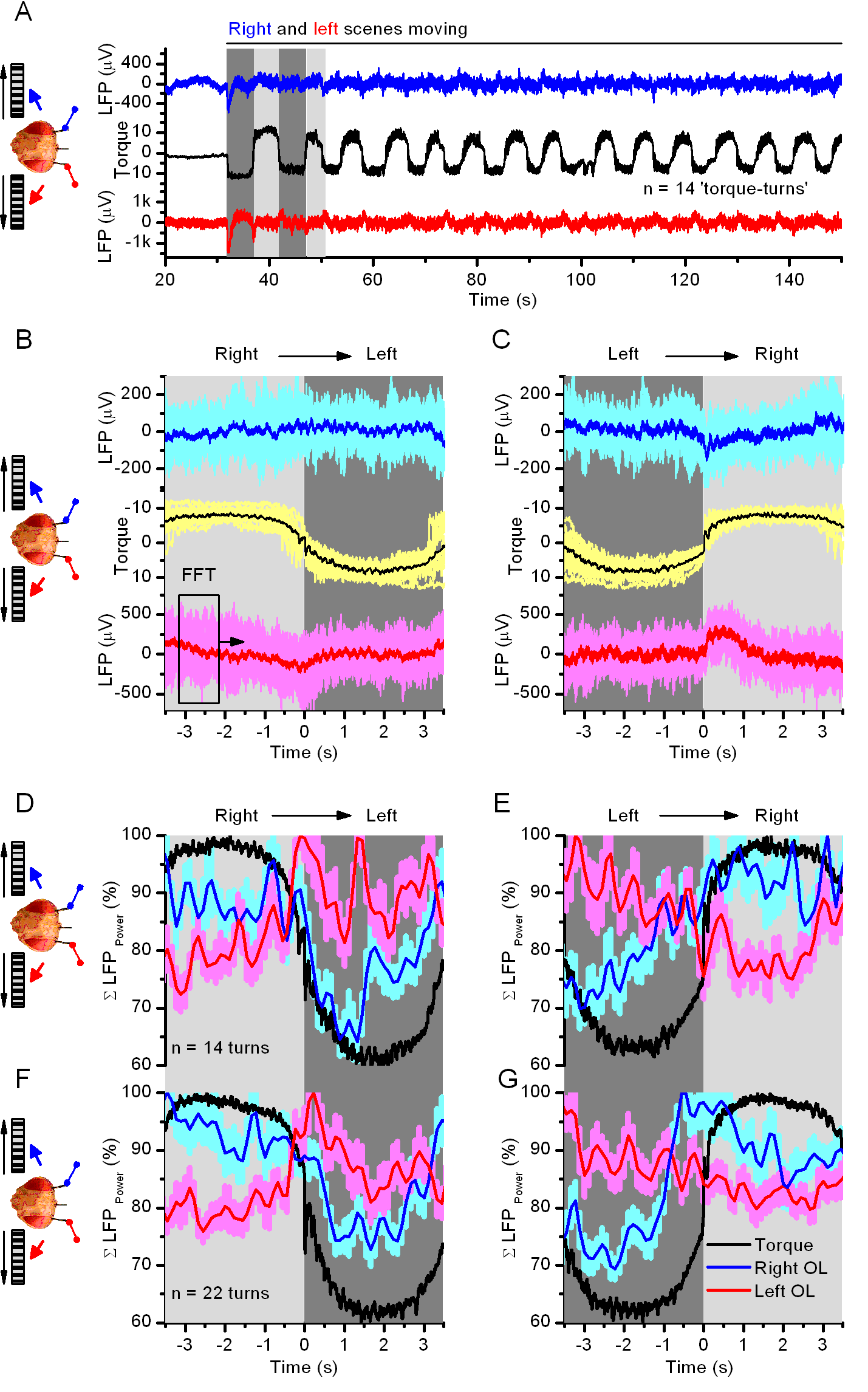

Supplement: Figure S10 — Intrinsic modulation of neural activity in the optic lobes during the competing stimuli paradigm; visual motion input to the eyes remains unchanged, yet the neural outputs of the optic lobes oppose each other. Changes in the mean neural output of the left and right optic lobes (OL), monitored as the summed power (20–100 Hz) of their LFPs, coincide with the torque responses. (A) LFPs from the right (blue) and left (red) optic lobes; yaw torque (black) shows attempts to rotate to right (up) and left (down), highlighted by light-gray and gray bars, respectively; data from the same fly as in Fig. S9, again with 14 torque responses to left and right. (B–C) shows neural activity during right-to-left and left-to-right choices. 14 LFPs from the right (cyan) and left (magenta) optic lobes superimposed and aligned by the corresponding zero-crossings in the torque (yellow). The means are shown in blue, red and black, respectively. (D–E) Power indexes of LFPs are then calculated for each separate torque response; here, giving 14 power-indexes for left and 14 power-indexes for right choices. For, each torque response we used a 1,000-point sliding window with 100-point jumps (B) and their frequencies from 20–100 Hz are summed up for each data-point. The mean ± SEM of these 14 power-indexes are shown in (D) and (E) for right-to-left and left-to-right choices, respectively. Changes in the output of the left and right optic lobes precede the changes in the torque responses, oscillating with a 180o phase shift. (F–G) shows similar dynamics in the power-indexes of another experiment (Fig. 6). (F: mean ± SEM). These general dynamics in LFPs are also seen in the pooled data for five flies, as shown in Fig. 9B. (9.84 MB TIF) [file pone.0014455.s011.tif]
